# Plexin-B1 Mutation Drives Metastasis in Prostate Cancer Mouse Models

**DOI:** 10.1158/2767-9764.CRC-22-0480

**Published:** 2023-03-16

**Authors:** Boris Shorning, Neil Trent, David F. Griffiths, Thomas Worzfeld, Stefan Offermanns, Matthew J. Smalley, Magali Williamson

**Affiliations:** 1 European Cancer Stem Cell Research Institute, School of Biosciences, Cardiff University, Cardiff, United Kingdom.; 2 Department of Cellular Pathology, University Hospital of Wales, Cardiff, United Kingdom.; 3 Institute of Pharmacology, University of Marburg, Marburg, Germany.; 4 Department of Pharmacology, Max-Planck-Institute for Heart and Lung Research, Bad Nauheim, Germany.; 5 School of Cancer and Pharmaceutical Sciences, Faculty of Life Sciences & Medicine, King's College London, London, United Kingdom.

## Abstract

Metastatic prostate cancer is essentially incurable and is a leading cause of cancer-related morbidity and mortality in men, yet the underlying molecular mechanisms are poorly understood. Plexins are transmembrane receptors for semaphorins with divergent roles in many forms of cancer.

We show here that prostate epithelial cell–specific expression of a mutant form of Plexin-B1 (*P1597L*) which was identified in metastatic deposits in patients with prostate cancer, significantly increases metastasis, in particular metastasis to distant sites, in two transgenic mouse models of prostate cancer (*PbCre^+^Pten^fl^^/fl^Kras^G12V^*and *PbCre^+^Pten^fl^^/fl^p53^fl/^^fl^*). In contrast, prostate epithelial cell–specific expression of wild-type (WT) Plexin-B1 in *PbCre^+^Pten^fl^^/fl^Kras^G12V^* mice significantly decreases metastasis, showing that a single clinically relevant *Pro1597Leu* amino-acid change converts Plexin-B1 from a metastasis-suppressor to a metastasis-promoter. Furthermore, *PLXNB1^P1597L^* significantly increased invasion of tumor cells into the prostate stroma, while *PLXNB1^WT^*reduced invasion, suggesting that Plexin-B1 has a role in the initial stages of metastasis. Deletion of RhoA/C or PDZRhoGEF in *Pten^fl^^/fl^Kras^G12V^PLXNB1^P1597L^* mice suppressed metastasis, implicating the Rho/ROCK pathway in this phenotypic switch*.* Germline deletion of Plexin-B1, to model anti-Plexin-B1 therapy, significantly decreased invasion and metastasis in both models.

Our results demonstrate that Plexin-B1 plays a complex yet significant role in metastasis in mouse models of prostate cancer and is a potential therapeutic target to block the lethal spread of the disease.

**Significance:**

Few therapeutic targets have been identified specifically for preventing locally invasive/oligometastatic prostate cancer from becoming more widely disseminated. Our findings suggest Plexin-B1 signaling, particularly from the clinically relevant *P1597L* mutant, is such a target.

## Introduction

Metastasis is the primary cause of morbidity and mortality in prostate cancer, yet despite increasing interest in treating oligometastatic disease with curative intent (1), few effective treatments specifically developed to counter the metastatic process are currently available in the clinic. It is also not clear what drives prostate cancer progression from locally advanced to invasive/disseminated disease but it is likely to result from the activation of a complex combination of multiple signaling pathways (2). Studies using *in vivo* models of prostate cancer of different genetic backgrounds allow the role of individual pathways contributing to the process to be defined and potential therapeutic targets identified.

The early stages of metastasis involve the migration and invasion of tumor cells out of the primary tumor through the basement membrane and beyond. One set of genes implicated in this process is plexins, cell surface receptors for semaphorins (3). Vertebrates possess nine plexin genes, classified into four subfamilies [class A(1–4), B(1–3), C1, and D1] (3). Plexin stimulation delivers directional cues for cell migration and axon guidance through the regulation of several small GTPases and semaphorin-plexin signaling can either be attractive or repulsive depending on the particular plexin coreceptor expressed (4, 5), while nonpolarized stimulation of cells with semaphorins results in cell collapse (6). B-class plexins can interact with the GTPases Rnd1–3 (7, 8), Rac (9), RhoD (10), R-Ras (11), and M-Ras (12) and regulate Rho via PDZRhoGEF/LARG (13) and p190RhoGAP (14). In addition, the plexin cytoplasmic tail contains a GTPase-activating protein (GAP) domain (15) divided into two regions by a Rho binding domain (16) and plexins act as GAPs for Rap1B and Rap2A (17). Plexin-B1 interacts with the receptor tyrosine kinase receptors ErbB2 (18) and c-Met (19) and regulates the translocation of the hormone receptors androgen receptor (AR) or glucocorticoid receptor to the nucleus where they are active (20, 21). Loss of Plexin-B1 expression is associated with poor prognosis in melanoma (22) and estrogen receptor–positive breast cancer (23) but is a favorable prognostic factor for ErbB2-amplified breast cancer (24) and ovarian cancer (25) suggesting that Plexin-B1 can act as a tumor suppressor or an oncogene depending on context.

Somatic missense mutations in Plexin-B1 have been detected in patient samples of prostate cancer metastases (26). Recent studies have shown a *PLXNB1* mutation rate of around 5% [ranging from 3.3% (27), 3.4% (28), 4.9% (29), to 7.8% (30)] (cBioportal), compared with 9.8%, 6.8%, 2.4%, and 13.7% in PTEN in the same studies. Functional analysis *in vitro* of three such mutations (T1697A, T1795A, L1815P), demonstrated that these sequence changes inhibit the interaction of Rnd1, Rac, and R-Ras with Plexin-B1 and block the ability of Plexin-B1 to mediate R-Ras inactivation (26, 31). Overexpression of either of these three mutations, or of another GAP domain mutation (*P1597L*; Supplementary Fig. S1A), increased cell motility, invasion, and anchorage-independent growth of prostate cancer cells *in vitro*. In contrast, overexpression of WT Plexin-B1 reduced cell motility and invasion (26). The *P1597L* mutation, investigated in this study, was found in three prostate cancer bone metastases and two lymph node metastases, while a different mutation, at the same site (*P1597S*) was found in three lymph node metastases (26). A better understanding of the role of mutant Plexin-B1 in metastatic prostate cancer, and the downstream pathways it activates, offers the potential for novel therapeutic approaches in this setting.

To gain *in vivo* insights into the cellular mechanisms involved in metastasis and to investigate the role of Plexin-B1 in this process, we genetically manipulated the expression of WT and mutant (*P1597L*) Plexin-B1 in two models of prostate cancer and studied the effect on prostate cancer progression *in vivo*. The two mouse models were chosen to recapitulate common defects found in prostate tumors (32), namely: loss of PTEN or dysregulation of PI3K/Akt pathway, Ras/MAPK activation and/or p53 loss or mutation. We find that Plexin-B1 plays a significant role in the spread of prostate cancer, with high expression of the clinically relevant *P1597L*-mutant enhancing metastasis but expression of the WT protein suppressing it, likely through activation of the Rho/ROCK (Rho-associated protein kinase) pathway. This pathway has potential as a therapeutic target in locally advanced or oligometastatic prostate cancer, particularly when mutant Plexin-B1 is present.

## Materials and Methods

### Experimental Animals

This study was approved by the Cardiff University Animal Welfare and Ethical Review Body and carried out under the authority of appropriate Home Office Personal and Project Licences and with reference to ARRIVE guidelines. Animals were monitored regularly and predefined humane endpoints were strictly adhered to (33). Research was hypothesis and objective driven to minimize the number of animals used but power calculations ensured sufficient animals were included in cohorts to gain statistically significant results. Randomization was not appropriate as animals had to be assigned to cohorts according to their genotype. *Pb-Cre^+^ (Pb-Cre4, ARR2PB)* mice were obtained from the Mouse Models of Human Cancers Consortium (NCI, Frederick, MD). The *PB-Cre* transgene was incorporated into cohorts using male mice, as *PB-Cre^+^* female mice have been shown to recombine in the ovaries (34, 35). Littermate controls lacking the *Pb-Cre* transgene were used in all experiments. Mice homozygous for floxed *Pten* exons 4 and 5 (*Pten^fl^^/^^fl^*; ref. 36), mice carrying inducible endogenous *Kras^G12V^* oncogene (37), mice homozygous for floxed *p53* exons 2–10 (*p53^fl/^^fl^*; ref. 38) and constitutive *Plexin-B1*–knockout (*PlxnB1^−^^/^^−^)* mice lacking exons 13–16 (39) have been described previously. Mice were maintained on an outbred background. RhoA^flox/flox^, RhoC^Δ2–3^, and PDZRhoGEF^−/−^ mice were obtained from Yi Zheng lab.

### Genetically Modified Mice

Mice carrying a conditionally activated knock-in construct of human Plexin-B1 containing WT or a C5060T mutation causing a substitution of proline 1597 to leucine (*loxP-STOP-loxP-PLXNB1^WT^* or *loxP-STOP-loxP-PLXNB1^P1597L^* mice; hereafter *PLXNB1^WT^* and *PLXNB1^P1597L^*) were commercially generated by genOway. The cDNA construct *P1597L-PLXNB1* had been made and sequenced previously (26). A transgenic cassette expressing the WT or mutated *P1597L-PLXNB1 cDNA – hGH polyA* under the control of the *CAG* promoter and with a neomycin STOP sequence flanked by two *loxP* sites was generated. GenOway's validated *Rosa26* “Quick Knock-in” approach was then used to introduce a single copy of the cassette into the *Rosa26* locus on chromosome 6 through homologous recombination in embryonic stem (ES) cells (Supplementary Fig. S1). The linearized construct was transfected into mouse 129Sv ES cells according to standard electroporation procedures (i.e., 5 × 10^6^ ES cells in presence of 40 μg of linearized plasmid, 260 V, 500 μF). Positive selection was started 48 hours after electroporation by addition of 200 μg/mL of G418. G418-resistant clones were screened for the correct homologous recombination event at the *Rosa26* locus by PCR and southern blotting. Three correctly recombined ES clones were used for injection into C57BL/6J blastocysts. Injected blastocysts were reimplanted into OF1 pseudopregnant females and allowed to develop to term. After approximately 3 weeks, a total of 3 male chimeric mice were produced per construct with a chimerism rate above 50%. These animals were mated with WT C57BL/6J females to generate heterozygous mice carrying the *Rosa26* floxed allele in their germline.

To assess whether the ES cells contributed to the germ layer of the chimeras, mouse coat color markers were used. The coat color marker of the 129Sv ES cells (agouti) is dominant over the black coat color of the C57BL/6 mice. Agouti F1 progeny were screened for the *Rosa26* knock-in allele by PCR and southern blotting, using genomic DNA isolated from tail biopsies. A total of 9 animals out of 30 carried the allele and 6 animals were verified further by southern blotting (Supplementary Fig. S1).

*PLXNB1^WT^* and *PLXNB1^P1597L^* knock-in animals were bred to *Pb-Cre^+^* mice to obtain animals with prostate epithelial–specific expression of WT or mutant Plexin-B1 protein. Mice were genotyped for the *PLXNB1^WT^* or *PLXNB1^P1597L^*-inducible allele (heterozygous or homozygous) by PCR either according to a genOway protocol (forward *AAGACGAAAAGGGCAAGCATCTTCC*, reverse *GCAGTGAGAAGAGT ACCACCATGAGTCC*, 94°C for 2 minutes and 35 cycles of 94°C for 30 seconds, 65°C for 30 seconds, 68°C for 5 minutes, giving a 1,870 bp product in the inducible *PLXNB1^P1597L^* mouse) or according to our simplified protocol identical to quantitative RT-PCR analysis of *PlxnB1* expression. To distinguish between heterozygous and homozygous *PLXNB1^P1597L^* we followed a protocol developed by genOway (forward *CAATACCTTTCTGGGAGTTCTCTGC*, reverse *CTGCATAAAACCCCAGATGACTACC*, 94°C for 2 minutes and 35 cycles of 94°C for 30 seconds, 55°C for 30 seconds, 72°C for 30 seconds, giving a 304 bp product in WT or heterozygous *PLXNB1^P1597L^* mouse and no product in homozygous *PLXNB1^P1597L^* mouse).

### Histologic Analysis and IHC

Primary tumors were excised and weighed (Supplementary Table S1). Prostate tissue was dissected in 1× PBS and fixed in ice-cold 10% neutral buffered formalin for no longer than 24 hours before being processed into paraffin blocks according to standard procedures. For IHC, 5 μm sections were dewaxed in xylene, rehydrated in ethanol, and antigen retrieval was performed by heating in either citrate (pH 6.0) or Ethylenediaminetetraacetic acid (EDTA) buffer (pH 8.0) in a pressure cooker for 15 minutes after reaching full pressure. Sections were cooled for 15 minutes, blocked in 0.5% hydrogen peroxide for 5 minutes at room temperature, and then blocked with 20% normal rabbit or goat serum (DAKO, Agilent) for 20 minutes and incubated with the primary antibody overnight at +4°C. After washing in TBS/0.05% Tween, sections were incubated in secondary antibody for 30 minutes (EnVision+ System- HRP Labeled Polymer; Dako) and the staining was visualized with DAB (EnVision+ System). Details for all the primary antibodies used in study are listed in Supplementary Table S2. Tissue sections were assessed by a pathologist with a special interest in urological pathology and on the specialist register who was masked to the genotype. Prostate epithelial cells were visualized by AR and pan-cytokeratin staining.

*Ki67*, phospho-MLC2^Ser19^, and AR staining were each quantified in prostates of 100-day-old mice (for each stain *n* = 3 per cohort, 5 fields for each sample). *Ki67* staining was scored “blind” as positive or negative and compared with the total number of nuclei in a field. For semiquantitative analysis of phospho-MLC2^Ser19^ staining, we used a Histo-score (H-score) formula: 3 × percentage of strongly staining cells + 2 × percentage of moderately staining cells + percentage of weakly staining cells, giving a range of 0 to 300, scored “blind.”

For quantitation of invasion, 5–10 random images of tumor sections, from each of three biological replicates, were scored blind for invasion into the stroma, at 40× magnification. Invasion was scored by counting pan-cytokeratin positive cells breaking the basement membrane or located inside the stromal compartment, divided by total number of pan-cytokeratin positive cells.

### RNA Extraction and qRT-PCR Analysis

Prostate lobes were dissected in ice-cold PBS. Tissues were homogenized in TRIzol Reagent (Invitrogen), extracted using standard phenol–chloroform protocol and RNA was purified further using an RNA extraction kit (Norgen). RNA from dorsolateral lobes of age-matched 100-day-old mice (*n* = 2 of each genotype) were used for qRT-PCR. Reverse transcription was performed using the SuperScript III reverse transcriptase kit and random hexamers (Invitrogen) according to the manufacturer's instructions. SYBR Select Master Mix (Applied Biosystems, Thermo Fisher Scientific) was added to cDNA samples and primers. Samples were run using QuantStudio 6 Flex Real-Time PCR System (Applied Biosystems). Reverse transcriptase negative controls were included in all analyses. Plexin-B1 primers were used for detecting both endogenous *PlxnB1* and *PLXNB1^MUT^*transcripts (forward-*TGTCACTATCAGGGGCTCCA*, reverse-*CTCCCCGCTGGCTCCAGTGAT*, 94°C for 2 minutes and 35 cycles of 94°C for 30 seconds, 55°C for 30 seconds, 72°C for 30 seconds, giving 145 bp products for both *WT* and inducible *PLXNB1^MUT^*). *β-Actin* and *GAPDH* were used as reference genes.

### Data Availability

Data available on request.

## Results

### Establishing Mouse Models of Plexin-B1 Overexpression

To understand the contribution of the clinically relevant Plexin-B1(*P1597L*) mutation to prostate cancer progression, we established two lines of mice carrying a targeted insertion of either *PLXNB1^WT^* or *PLXNB1^P1597L^* cDNA preceded by a *flox-STOP-flox* cassette into the *Rosa26* locus. Targeting was confirmed by Southern blotting (Supplementary Fig. S1). Activation/overexpression of these conditional alleles in the prostate was achieved by crossing with a line in which CRE recombinase was expressed under the *Probasin* promoter, which is specifically expressed in the prostate epithelium only (see Materials and Methods). Expression of Plexin-B1 in prostates of *PLXNB1^WT^* and *PLXNB1^P1597L^* mice was compared with unmanipulated WT mice and mice with a germline deletion of Plexin-B1 (*PlxnB1^−^^/^^−^*; ref. 39; Supplementary Fig. S2). Plexin-B1 protein was expressed in the epithelial cells of all lobes of WT mouse prostates, localizing to the cell membrane, cytoplasm, and nucleus of prostate epithelial cells, but was absent from prostate stroma (Supplementary Fig. S2) and was absent in *PlxnB1^−^^/^^−^* mice. Similar levels of high expression of Plexin-B1 were observed in prostate epithelial cells of *PLXNB1^WT^* and *PLXNB1^P1597L^* mice (Supplementary Fig. S2). No obvious structural or histologic changes in the prostate were found between WT and *PLXNB1^WT^* or *PLXNB1^P1597L^* mice. Mice from all lines were fertile and survived over 500 days.

Prostate tumors are characterized by loss of PTEN and dysregulation of the PI3K/AKT pathway (32, 40), p53 deletion or mutation (41), and activation of the Ras/Raf pathway (40). To determine whether Plexin-B1 contributes to prostate cancer progression *in vivo*, we tested the effect on tumor growth and metastasis of manipulating Plexin-B1 expression in two different transgenic mouse models of prostate cancer which recapitulate these common genetic alterations and which also metastasize: *PbCre^+^Pten^fl^^/fl^Kras^G12V^* (ref. 42; hereafter abbreviated to *Pten^fl^^/fl^Kras^G12V^*) and *PbCre^+^Pten^fl^^/fl^p53^fl/^^fl^* (ref. 43; hereafter abbreviated to *Pten^fl^^/fl^p53^fl/^^fl^*). Both the *Pten^fl^^/fl^Kras^G12V^* and *Pten^fl^^/fl^p53^fl/^^fl^* models have moderate/low metastatic ability that enables the analysis of additional alleles which may accelerate tumor formation/progression; prostate tumors in both models can metastasize to lumbar lymph nodes with limited ability to form distant metastases (42, 43). *Pten^fl^^/fl^Kras^G12V^* differs from the previously described highly metastatic *PbCre^+^Pten^fl^^/fl^Kras^G12D^* model (42) in using a less aggressive *Kras^G12V^* (37). The *Pten^fl^^/fl^Kras^G12V^* and *Pten^fl^^/fl^p53^fl/^^fl^* lines were crossed with *PLXNB1^P1597L^* mice. In addition, the *Pten^fl^^/fl^Kras^G12V^* line was crossed with *PLXNB1^WT^* mice to establish the effect of overexpression of WT Plexin-B1 on tumor progression.

Two cohorts were established for each cross, one for euthanasia at a fixed timepoint of 100 days and one for euthanasia when required for welfare reasons due to tumor morbidity. Primary prostate tumors, local lymph nodes, and visceral organs were all processed for histologic analysis to assess primary tumor morphology, extent of local invasion, and the presence of local or distant metastases.

### *PLXNB1^P1597L^* Overexpression Increases Survival of *Pten^fl/fl^Kras^G12V^* Mice

Plexin-B1 was expressed at moderate levels in the epithelial cells of *Pten^fl^^/fl^Kras^G12V^* prostate tumor cells (Supplementary Fig. S2E, antibody specificity shown in Supplementary Fig. S2F), and expressed at high levels in *Pten^fl^^/fl^Kras^G12V^PLXNB1^WT^* (Supplementary Fig. S2G and S2H) and *Pten^fl^^/fl^Kras^G12V^PLXNB1^P1597L^* primary tumors and metastases (Supplementary Fig. S2I–S2K).

**FIGURE 1:**
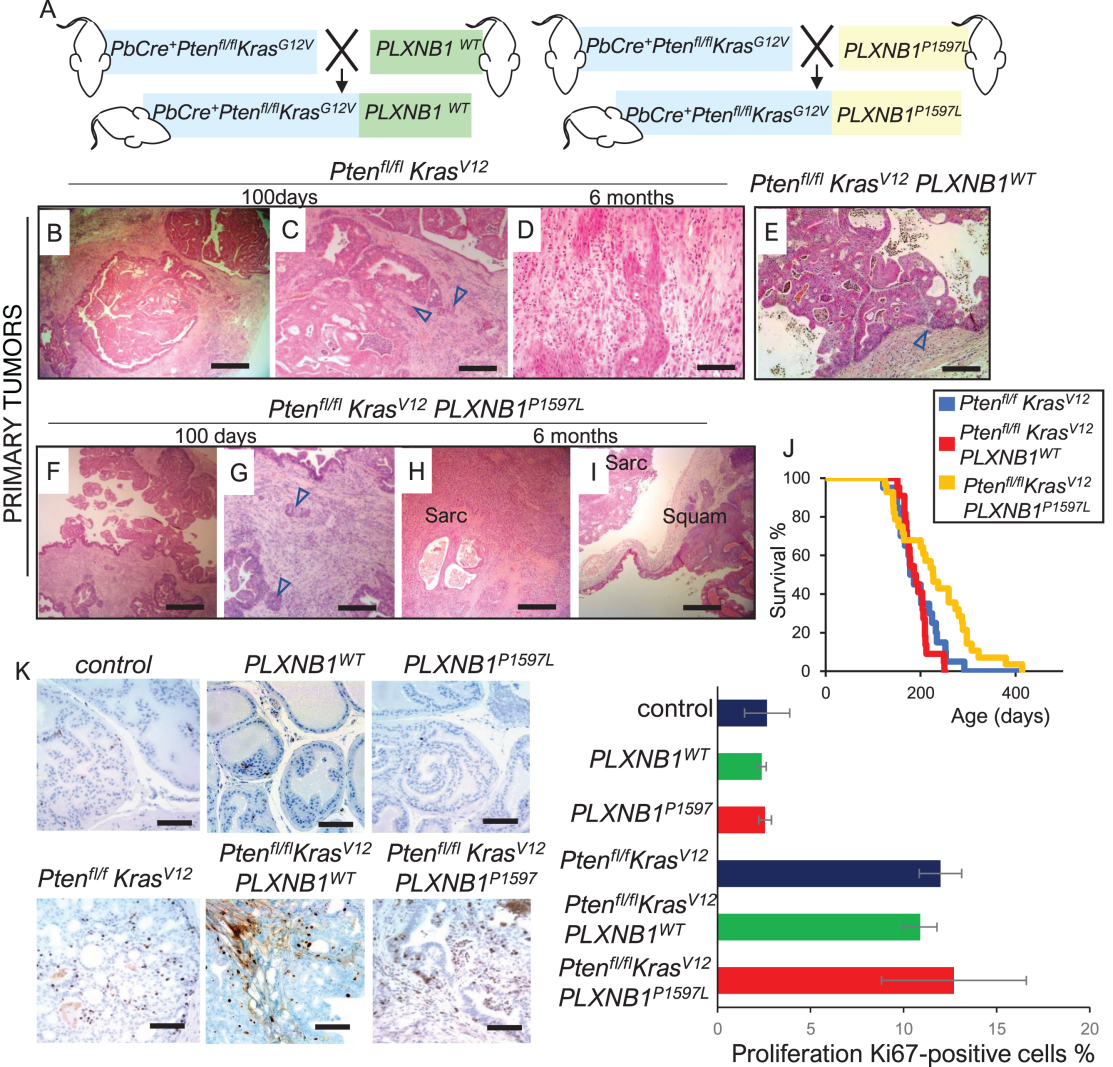
*PLXNB1^P1597L^* expression suppresses prostate tumor proliferation and extends survival in *Pten^fl/fl^Kras^G12V^* mice. **A,** Schematic diagram of generation of *Pten^fl/fl^Kras^G12V^PLXNB1^WT^* and *Pten^fl/fl^p53^fl/fl^PLXNB1^P1597L^* mice. **B–I,** Hematoxylin and eosin (H&E) histology of primary prostate tumors. **B** and **C,** Invasive adenocarcinoma in *Pten^fl/fl^Kras^G12V^* prostates at day 100 timepoint before the onset of metastasis; invasive tumor tissue is marked with triangular arrows [scale bar: 500 μm (**B**), 200 μm (**C**)]. **D,** Ages *Pten^fl/fl^Kras^G12V^* prostate (6–7 months) showing region of sarcomatoid carcinoma (scale bar: 200 μm). **E,***Pten^fl/fl^Kras^G12V^PLXNB1^WT^* prostate, signs of invasion were rare (marked with an arrow) in this cohort and stromal reaction was diminished (scale bar: 200 μm). **F** and **G,** Widespread invasion of glandular neoplastic cells (marked with arrows) into the stroma in *Pten^fl/fl^Kras^G12V^PLXNB1^P1597L^* prostates at the day 100 timepoint [scale bar: 500 μm (**F**), 200 μm (**G**)]. **H** and **I,** Heterogeneous prostate tumors in ages (>6 months old) *Pten^fl/fl^Kras^G12V^PLXNB1^P1597L^* mouse showing sarcomatoid phenotype (**H**, scale bar: 500 μm) or a mixture of adenomatous, sarcomatoid, and squamous phenotypes (**I**, scale bar: 500 μm)]. **J,** Kaplan–Meier survival curves for *Pten^fl/fl^Kras^G12V^* (*n* = 20), *Pten^fl/fl^Kras^G12V^PLXNB1^WT^* (*n* = 22), and *Pten^fl/fl^Kras^G12V^PLXNB1^P1597L^* (*n* = 28) cohorts. Primary prostate tumor growth was the major reason for euthanasia. The increase in survival of the *Pten^fl/fl^Kras^G12V^PLXNB1^P1597L^* cohort (median survival 226.5 days) compared with the *Pten^fl/fl^Kras^G12V^* cohort (median 182 days) is significant (log-rank test: *z* = 2.44, *P* = 0.0148, 95% confidence interval). **K,***Ki67* antigen staining and quantitation of proliferation rates for prostate epithelium of 100-day-old mice from control, *PLXNB1^WT^*, *PLXNB1^P1597L^*, *Pten^fl/fl^Kras^G12V^*, *Pten^fl/fl^Kras^G12V^PLXNB1^WT^*, and *Pten^fl/fl^Kras^G12V^PLXNB1^P1597L^* mice.

As described previously (40), prostates from *Pten^fl^^/fl^Kras^G12V^*-based cohorts displayed distorted glandular structure with focal areas of microinvasion adjacent to reactive stroma and regions of sarcomatoid metaplasia (Fig. 1A–D). There were no overt differences in histology of primary tumors between the *Pten^fl^^/fl^Kras^G12V^, Pten^fl^^/fl^Kras^G12V^PLXNB1^WT^*, and *Pten^fl^^/fl^Kras^G12V^PLXNB1^P1597L^* models (Fig. 1B–I).

*PLXNB1^WT^* overexpression made no significant difference to survival of the *Pten^fl^^/fl^Kras^G12V^* line (Fig. 1J). Unexpectedly, however, expression of *PLXNB1^P1597L^* significantly increased the survival of *Pten^fl^^/fl^Kras^G12V^* mice (Fig. 1J). *Pten^fl^^/fl^Kras^G12V^PLXNB1^P1597L^* mice survived for a median of 226.5 days, compared with 182 and 176 days for *Pten^fl^^/fl^Kras^G12V^* and *Pten^fl^^/fl^Kras^G12V^PLXNB1^WT^* mice, respectively (*P* = 0.0148 vs. *Pten^fl^^/fl^Kras^G12V^*; Fig. 1J). Prostate tumor growth in *Pten^fl^^/fl^Kras^G12V^PLXNB1^P1597L^* animals was heterogeneous. Cell proliferation rates in *Pten^fl^^/fl^Kras^G12V^PLXNB1^WT^* tumors were also not statistically different from those of *Pten^fl^^/fl^Kras^G12V^* cohorts at 100 days, as demonstrated by *Ki67* staining of prostate epithelial cells (Fig. 1K). However, there was a wide variation in tumor cell *Ki67* staining between different *Pten^fl^^/fl^Kras^G12V^PLXNB1^P1597L^* mice at 100 days, suggesting that this cohort developed a heterogeneous mix of slowly and rapidly growing primary tumors which overall resulted in an increase in median survival.

### *PLXNB1^P1597L^* Overexpression Suppresses Proliferation of *Pten^fl/fl^p53^fl/fl^* Primary Mouse Prostate Tumors and Increases Survival

Expression levels of Plexin-B1 were moderate in *Pten^fl^^/fl^p53^fl/^^fl^* tumor cells (Supplementary Fig. S2L, antibody specificity shown in Supplementary Fig. S2M) and high in *Pten^fl^^/fl^p53^fl/^^fl^PLXNB1^P1597L^* primary tumors and metastases (Supplementary Fig. S2N–S2P).

**FIGURE 2:**
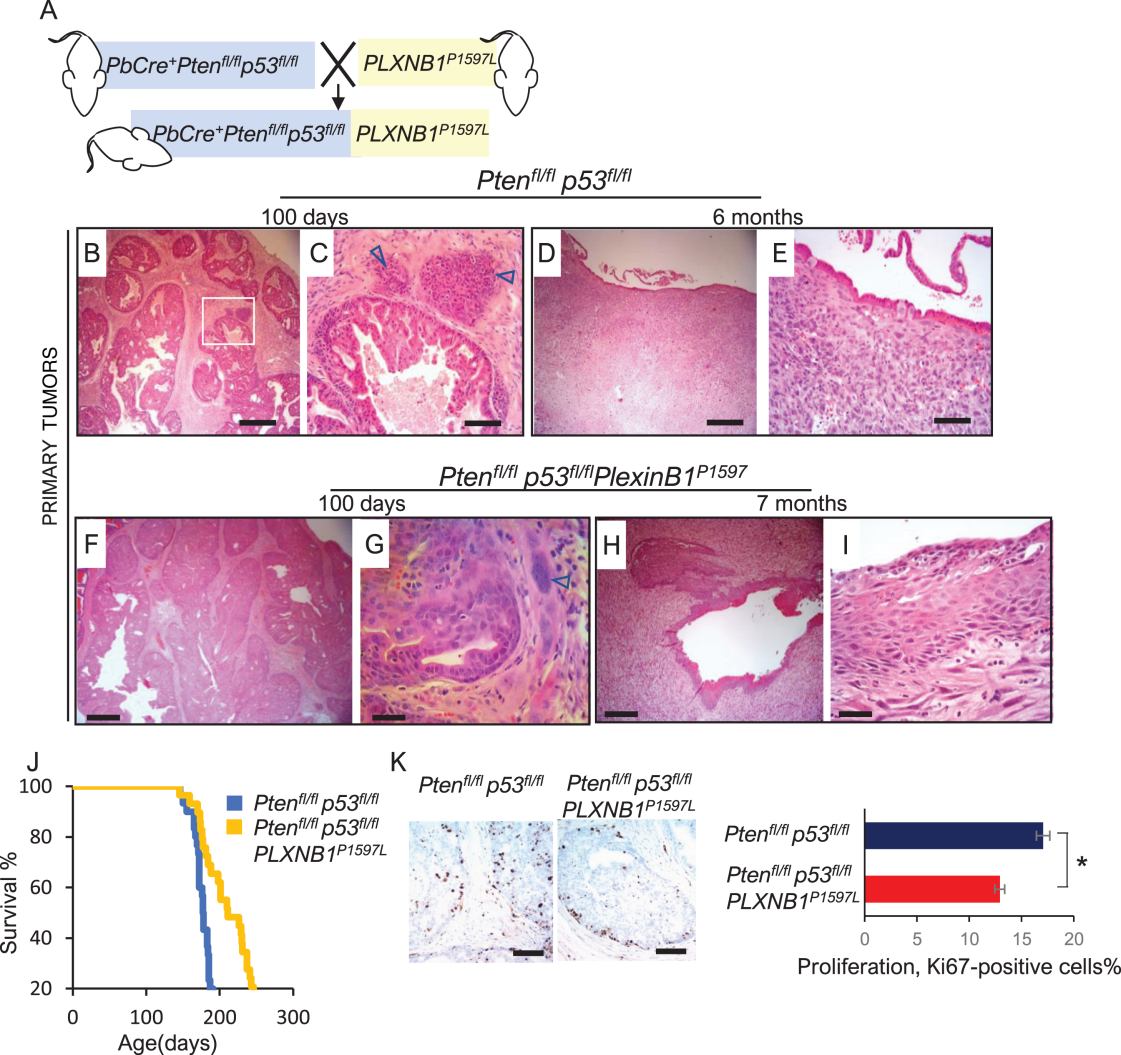
*PLXNB1^P1597L^* expression suppresses prostate tumor proliferation and extends survival in *Pten^fl^^/fl^p53^fl/^^fl^* mice. **A,** Schematic diagram of generation of *Pten^fl^^/fl^p53^fl/fl^PLXNB1^P1597L^* mice. **B–I,** H&E histology of primary prostate tumors in *Pten^fl^^/fl^p53^fl/^^fl^* cohorts. **B** and **C,** Invasive adenocarcinoma in *Pten^fl^^/fl^p53^fl/^^fl^* mouse prostate at day 100 timepoint showing sarcomatoid deposits next to the epithelium [marked with arrows; scale bar: 500 μm (**B**), 100 μm (**C**)]. **D** and **E,** Sarcomatoid carcinoma in prostates of 6-month-old *Pten^fl^^/fl^p53^fl/^^fl^* mice [scale bar: 500 μm (**D**), 50 μm (**E**)]. **F** and **G,** Invasive adenocarcinoma in mouse prostates at day 100 timepoint in *Pten^fl^^/fl^p53^fl/fl^PLXNB1^P1597L^* mice; sarcomatoid cells marked by arrows [scale bar: 500 μm (**F**), 50 μm (**G**)]. **H** and **I,** Widespread epithelial invasion into stroma and pronounced expansion of sarcomatoid mass combined with squamous differentiation of epithelium in prostates of 7-month-old *Pten^fl^^/fl^p53^fl/fl^PLXNB1^P1597L^* mice, scale bar: 500 μm (**H**), 50 μm (**I**). **J,** Kaplan–Meier survival curves for *Pten^fl^^/fl^p53^fl/^^fl^* (*n* = 30) and *Pten^fl^^/fl^p53^fl/fl^PLXNB1^P1597L^* (*n* = 29) cohorts. The increase in survival of the *Pten^fl^^/fl^p53^fl/fl^PLXNB1^P1597L^* cohort (median 211 days) compared with *Pten^fl^^/fl^p53^fl/^^fl^* (median 177 days) is significant (log-rank test, *z* = 4.86, *P* < 0.001, 95% confidence interval). **K,***Ki67* antigen staining and quantitation of proliferation rates for prostate epithelium of 100-day-old *Pten^fl^^/fl^p53^fl/^^fl^* and *Pten^fl^^/fl^p53^fl/fl^PLXNB1^P1597L^* mice *PLXNB1^P1597L^* expression suppressed proliferation in the *Pten^fl^^/fl^p53^fl/^^fl^* background. *, *P* < 0.05 (*t* test, *n* = 3, mean ± SD). Scale bars = 100 μm.

Prostate tumors from all *Pten^fl^^/fl^p53^fl/^^fl^*-based cohorts had a marked increase in mesenchymal phenotype with little epithelial component compared with that of *Pten^fl^^/fl^Kras^G12V^*-based cohorts (Fig. 2A–I) and these sarcomatoid tumors were the cause of morbidity in accordance with earlier data (43). Prostate tumors in *Pten^fl^^/fl^p53^fl/^^fl^* and *Pten^fl^^/fl^p53^fl/^^fl^PLXNB1^P1597L^* mice showed similar progression from adenocarcinoma at day 100 toward sarcomatoid metaplasia at 6 months (Fig. 2B–I).

Expression of *PLXNB1^P1597L^*significantly increased the survival of *Pten^fl^^/fl^p53^fl/^^fl^* mice (Fig. 2J). Median survival of *Pten^fl^^/fl^p53^fl/fl^PLXNB1^P1597L^* mice was 211 days, compared with 177 days in *Pten^fl^^/fl^p53^fl/^^fl^* mice (*P < 0.001;*Fig. 2J). Consistent with these findings, *Ki67* staining of prostates of 100-day *Pten^fl^^/fl^p53^fl/fl^PLXNB1^P1597L^* mice showing a 1.32-fold decrease in cell proliferation compared with the *Pten^fl^^/fl^p53^fl/f^* cohort (*P* < 0.01; Fig. 2K).

Prostate cancer mouse models typically need to be euthanized as a result of local complications associated with primary tumor bulk. The suppression of primary tumor proliferation by *PLXNB1^P1597L^* overexpression may contribute to the extended survival in these models.

### *PLXNB1^P1597L^* Significantly Increases Metastasis and *PLXNB1^WT^* Significantly Decreases Metastasis in the *Pten^fl/fl^Kras^G12V^* Mouse Model of Prostate Cancer

Next, we quantified the metastatic lesions in the different tumor cohorts by histologic examination. The prostate origin of metastatic lesions was confirmed by staining for AR, a prostate epithelial cell marker (Supplementary Fig. S3–S5).

**FIGURE 3:**
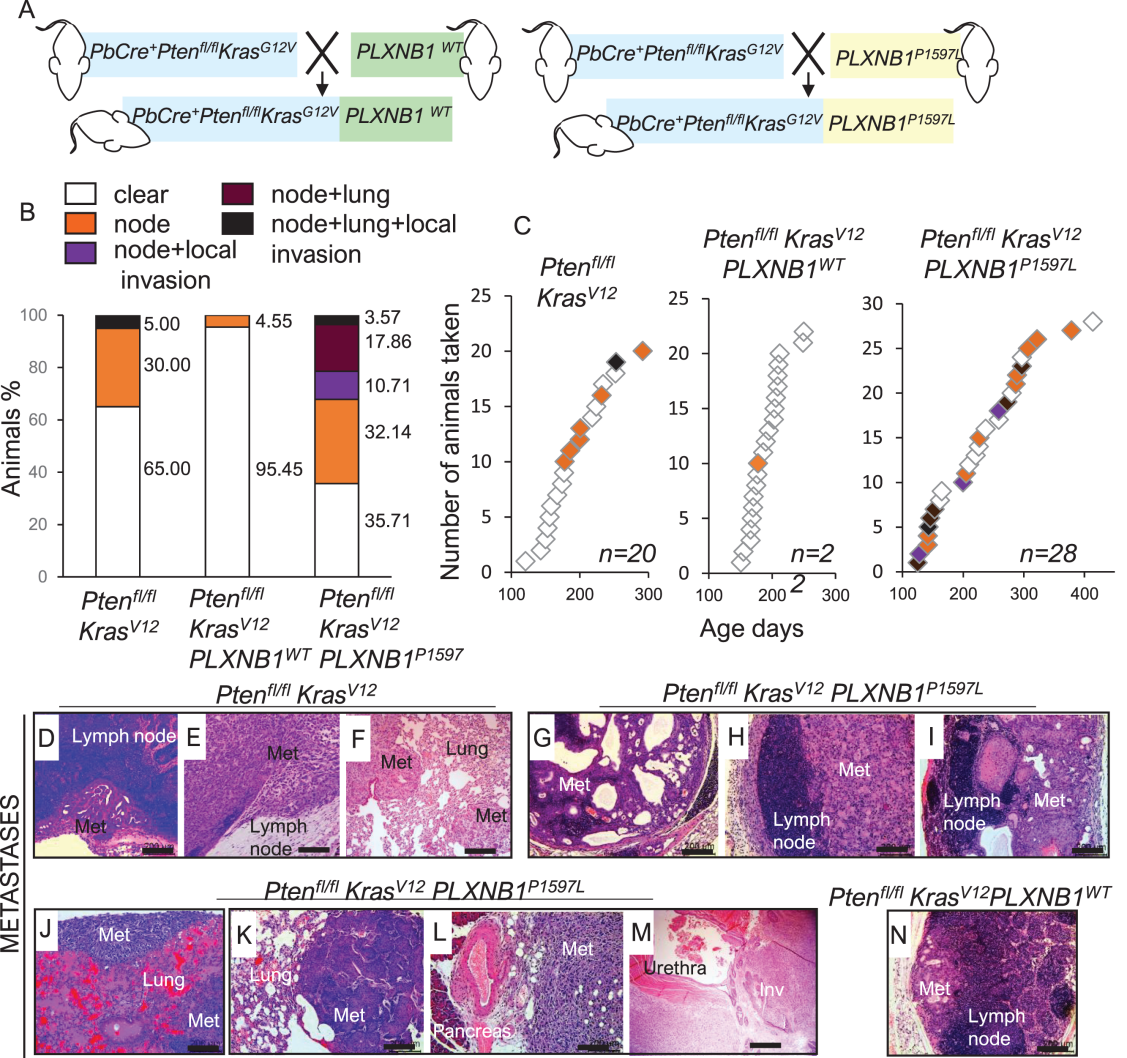
*PLXNB1^P1597L^* increases metastasis whereas *PLXNB1^WT^* expression suppresses metastasis in *Pten^fl^^/fl^Kras^G12V^* mouse models of prostate cancer (see also Supplementary Fig. S3–S5). **A,** Schematic diagram of crosses performed. **B,** Percentages of animals affected/not affected by metastasis in *Pten^fl^^/fl^Kras^G12V^* cohorts. Following necropsy, mice were categorized according to their metastatic outcome: no metastatic deposits (white), lymph node metastasis (orange), lymph node metastasis combined with invasion into peritoneum or pelvic muscle (purple), combined lymph node and lung metastasis (brown), animals with both lymph node and lung metastasis combined with invasion into peritoneum or pelvic muscle (black). **C,** Timing and type of metastatic deposits in *Pten^fl^^/fl^Kras^G12V^* cohorts. **D,** Typical epithelial gland-like metastasis in lymph node from *Pten^fl^^/fl^Kras^G12V^* cohort. Rare sarcomatoid nodules in lymph nodes (**E**) combined with sarcomatoid metastases in the lung (**F**) observed in a single mouse (of 20) in the *Pten^fl^^/fl^Kras^G12V^* cohort scale bar: 200 μm. Heterogeneous lumbar lymph node metastases from *Pten^fl^^/fl^Kras^G12V^PLXNB1^P1597L^* mice, including mixed epithelial/sarcomatoid deposits (**G**), sarcomatoid (**H**), and squamous metaplasia (**I**) [scale bar: 200 μm (**G**–**I**)]. Organ metastasis and local invasion in *Pten^fl^^/fl^Kras^G12V^PLXNB1^P1597L^* mice showing lung metastatic deposit with sarcomatoid (**J**) and squamous histology (**K**), abdominal metastasis adjoining pancreas (**L**) and prostate tumor invading urethra (**M**). Scale bar: 200 μm (**J**–**L**), 500 μm (**M**). **N,** The single lymph node deposit observed in the *Pten^fl^^/fl^Kras^G12V^PlxnB1^WT^*cohort (scale bar: 200 μm).

Importantly, expression of mutant *PLXNB1^P1597L^*in *Pten^fl^^/fl^Kras^G12V^* significantly increased the percentage of mice with metastases, compared with the parental line (*P* = 0.0452) and to *Pten^fl^^/fl^Kras^G12V^PLXNB1^WT^* mice (*P* < 0.0010; Fig. 3A–C; Table 1). This increase in metastases was particularly evident in sites distant from the prostate—the number of mice with lung metastases increased from 5% to 21.43% upon expression of *PLXNB1^P1597L^*.

**TABLE 1:** Number of mice with metastases

Genotype	Total mice	Local metastasis only		Mice with distant metastasis (lung/liver)		Total mice with metastases		*P* value *χ*^2^ versus Pten^fl/fl^Kras^G12V^	Statistical significance
	***N***	***n***	**%**	***n***	**%**	***n***	**%**		
*Pten^fl/fl^Kras^G12V^*	20	6	30	1	5	7	35		
*Pten^fl/fl^Kras^G12V^* *PlxnB1^−/−^*	28	3	10.71	0	0	3	10.71	0.0411	Significant (*P* < 0.05)
*Pten^fl/fl^Kras^G12V^* *PLXNB1^WT^*	22	1	4.55	0	0	1	4.55	0.0121	Significant (*P* < 0.05)
*Pten^fl/fl^Kras^G12V^* *PLXNB1^P1597L^*	28	11	39.3	7	25	18	64.29	0.0452	Significant (*P* < 0.05)
**Genotype**	**Total mice**	**Local metastasis only**		**Mice with distant metastasis (lung/liver)**		**Total mice with metastases**		***P* value *χ*^2^ versus Pten^fl/fl^p53^fl/fl^**	**Statistical significance**
	***N***	***n***	**%**	***n***	**%**	***n***	**%**		
*Pten^fl/fl^p53^fl/fl^*	30	4	13.33	0	0	4	13.33		
*Pten^fl/fl^p53^fl/fl^* *PlxnB1^−/−^*	21	0	0	0	0	0	0	0.081	Trend (*P* < 0.1)
*Pten^fl/fl^p53^fl/fl^* *PLXNB1^P1597L^*	29	12	41.38	0	0	12	41.38	0.0154	Significant (*P* < 0.05)

The metastatic deposit composition varied between adenocarcinoma, sarcomatoid (low pan-cytokeratin staining), and squamous areas (high pan-cytokeratin staining; Supplementary Fig. S3–S5). Adenocarcinoma was the predominant tissue type in *Pten^fl/fl^Kras^G12V^* cohort metastases (Fig. 3D–F; Supplementary Fig. S3) with one animal with a lymph node metastatic deposit of predominantly squamous differentiation at day 200 (Supplementary Fig. S3C) and one animal with sarcomatoid deposits in nodes, peritoneum and lung at day 253 (Fig. 3D–F; Supplementary Fig. S3F).

Metastases in *Pten^fl/fl^Kras^G12V^PLXNB1^P1597L^* mice showed a greater heterogeneity (Fig. 3G–M; Supplementary Fig. S4); 6 of 10 *Pten^fl/fl^Kras^G12V^PLXNB1^P1597L^* mice that were taken by day 200 developed predominantly sarcomatoid metastases (Fig. 3H, J, L; Supplementary Fig. S4A–F and S4H) and *Pten^fl/fl^Kras^G12V^PLXNB1^P1597L^* mice taken after day 200 displayed mixed adenocarcinoma/squamous differentiation (Fig. 3; Supplementary Fig. S4). There was no correlation between the increased age of *Pten^fl/fl^Kras^G12V^PLXNB1^P1597L^* mice and the presence of local or distant metastases (Supplementary Fig. S4). Mice without metastasis lived on average longer than those with distant metastasis, showing that the presence of metastases cannot be explained by an increase in survival.

In contrast, overexpression of *PLXNB1^WT^* significantly suppressed the metastatic spread of tumors in *Pten^fl/fl^Kras^G12V^* mice (*P* = 0.0121 and *P* < 0.001 compared with *Pten^fl/fl^Kras^G12V^* and *Pten^fl/fl^Kras^G12V^PLXNB1^P1597L^* mice, respectively; Fig. 3B and C; Table 1), with only a single local metastasis observed in *Pten^fl/fl^Kras^G12V^PLXNB1^WT^* mice (4.5%; Fig. 3N; Supplementary Fig. S5) of adenocarcinoma histology*.*

### *PLXNB1^P1597L^* Significantly Increases Metastasis in the *Pten^fl/fl^p53^fl/fl^* Mouse Model of Prostate Cancer

**FIGURE 4:**
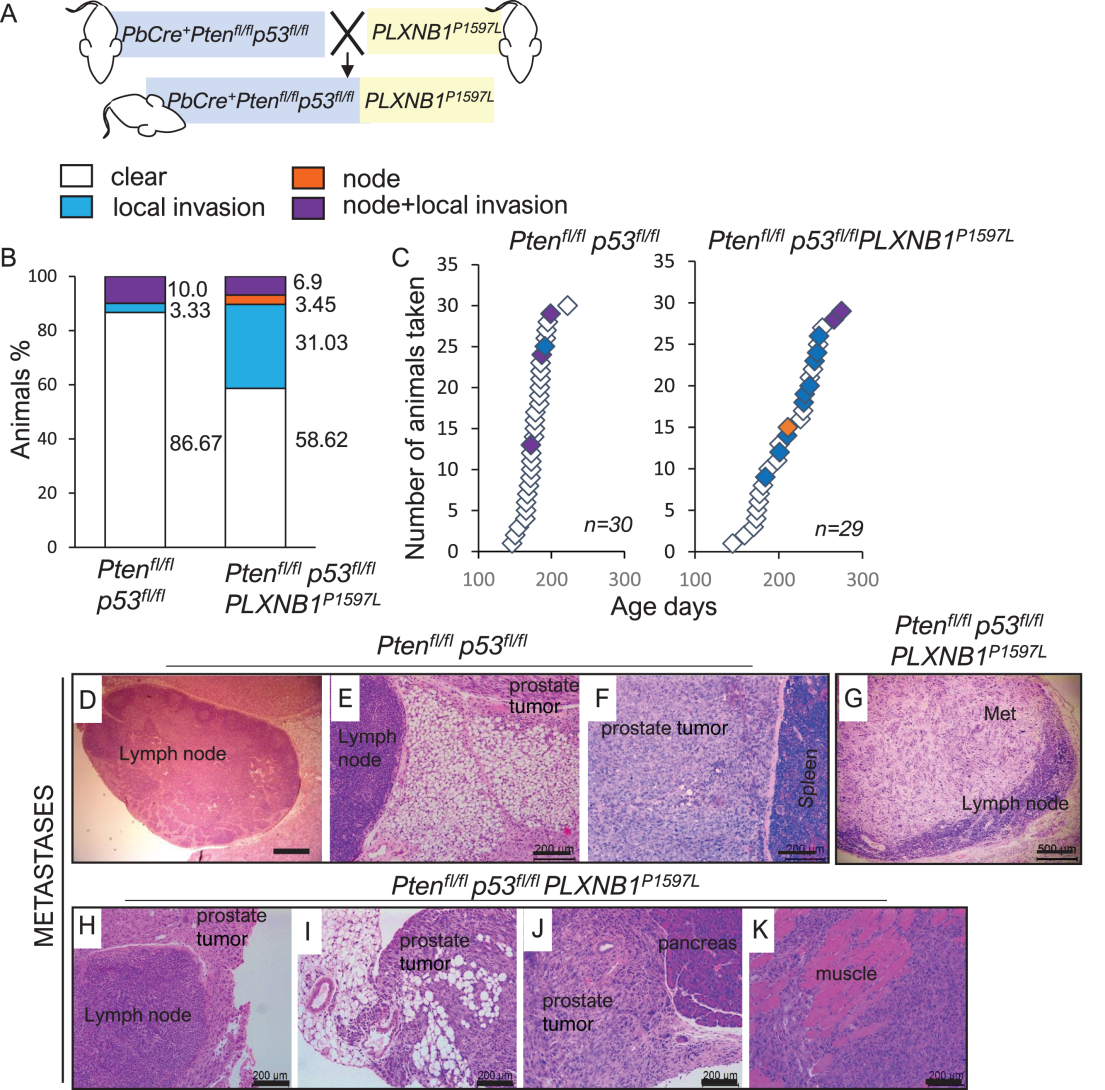
*PLXNB1^P1597L^* increases metastasis in *Pten^fl/fl^p53^fl/fl^* mouse models of prostate cancer (see also Supplementary Fig. S6 and S7). **A,** Schematic diagram of crosses performed. **B,** Percentage of animals affected/not affected by metastasis in *Pten^fl/fl^p53^fl/fl^* cohorts: no metastatic deposits (white), lymph node metastasis (orange), invasion into peritoneum or pelvic muscle (blue), lymph node metastasis combined with invasion into peritoneum or pelvic muscle (purple). **C,** Timing and type of metastatic deposits in *Pten^fl/fl^p53^fl/fl^* cohorts. See Table 1 for statistical analyses. Prostate sarcomatoid deposits on the perimeter of a lumbar lymph node (**D** and **E**) and adjoining spleen (**F**) in *Pten^fl/fl^p53^fl/fl^* mouse, scale bar: 500 μm (**D**), 200 μm (**E** and **F**). Metastatic deposits in lumbar lymph nodes (**G**) and on the node perimeter invading to peritoneum (**H**), peritoneum (**I**), pancreas (**J**), and sarcomatoid prostate tumor invading pelvic muscle (**K**) of *Pten^fl/fl^p53^fl/fl^PLXNB1^P1597L^* mice [scale bar: 500 μm (**G**), 200 μm (**H**–**K**)].

Expression of mutant *PLXNB1^P1597L^*in *Pten^fl/fl^p53^fl/fl^* mice significantly increased the percentage of mice with metastases, in comparison with the parental *Pten^fl/fl^p53^fl/fl^* line (*P* = 0.0154; Fig. 4A–C; Table 1). Metastatic deposits in *Pten^fl/fl^p53^fl/fl^* mice were all represented by the primary sarcomatoid tumors encroaching the lumbar lymph nodes and invading further into the peritoneum (Fig. 4D–F; Supplementary Fig. S6). *Pten^fl/fl^p53^fl/fl^PLXNB1^P1597L^* mice showed a marked increase in locally invasive tumors demonstrating invasion of the primary tumor into local structures or organs including peritoneum, pelvic or bladder muscle, vas deferens, and lymph node (Fig. 4G–K; Supplementary Fig. S7). The relationship between the presence of local metastasis and survival in *Pten^fl/fl^p53^fl/fl^PLXNB1^P1597L^* mice is shown in Supplementary Fig. S7M.

### *PLXNB1^P1597L^* Increases Local Invasion of Prostate Tumor Cells

Our results show that prostate epithelial cell–specific overexpression of mutant (*P1597L*) Plexin-B1 increases metastasis. Cancer metastasis is a multistep process which begins with tumor cell invasion from prostate acini through the basal membrane and into the prostate stroma. To establish the stage at which stage Plexin-B1 expression affects metastasis, we investigated the effect of expression of the different forms of Plexin-B1 on invasion of primary tumor cells into the prostate stroma.

**FIGURE 5:**
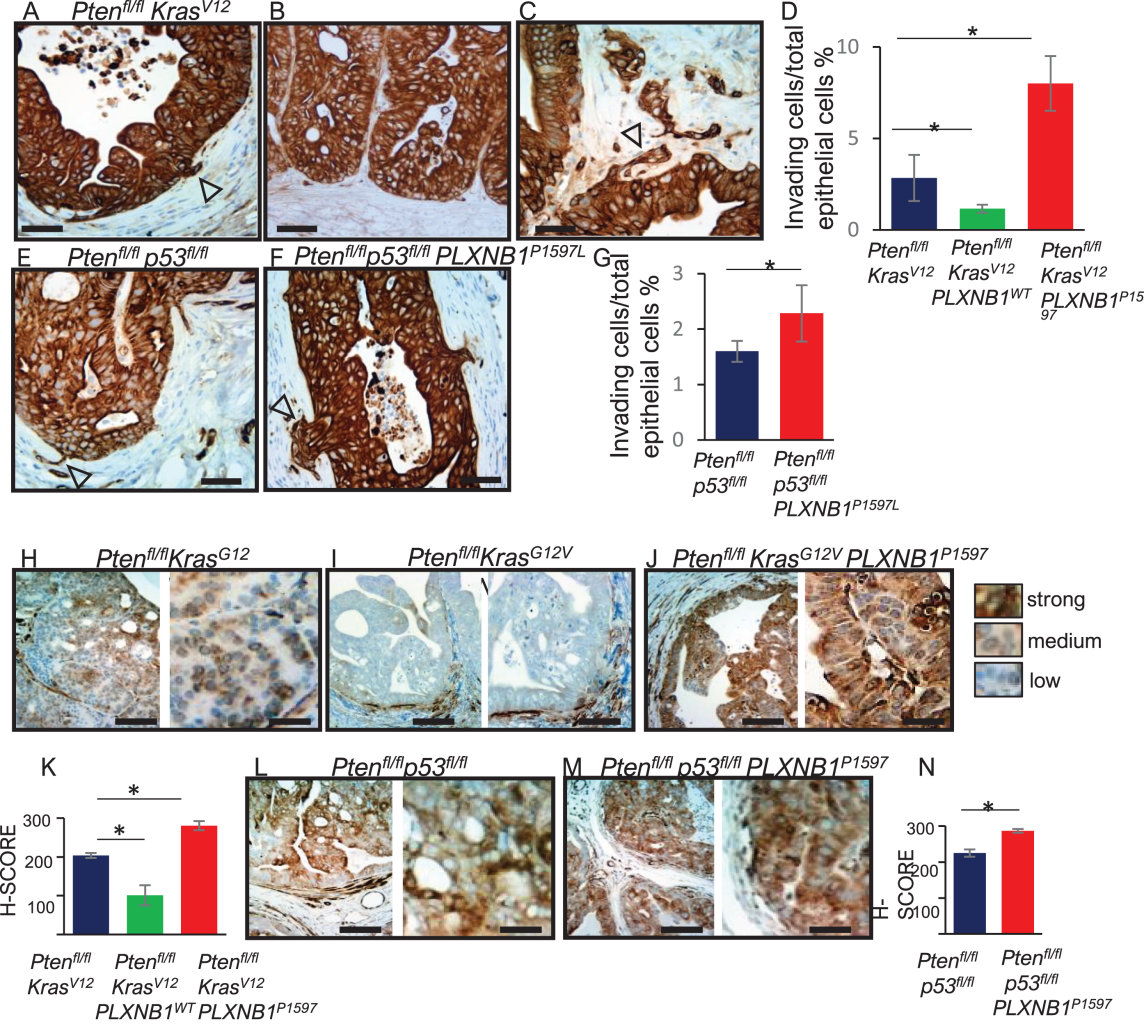
*PLXNB1^P1597L^* expression in the mouse prostate epithelium of *Pten^fl/fl^Kras^G12V^* and *Pten^fl/fl^p53^fl/fl^* mice promotes local invasion by prostate tumor cells and myosin phosphorylation. **A–F,** Immunostaining of prostates of 100-day-old mice with cytokeratin AE1/AE3 (pan-cytokeratin) to identify prostate epithelial cells breaking basement membrane and invading stroma in *Pten^fl/fl^ Kras^G12V^*(**A–C**) and *Pten^fl/fl^p53^fl/fl^* cohorts (**E** and **F**). Invading cells are indicated with arrowheads. Scale bars, 50 μm. Quantitation in the *Pten^fl/fl^Kras^G12V^* and *Pten^fl/fl^p53^fl/fl^* backgrounds shown in **D** and **G**, respectively. Pan-cytokeratin positive cells breaking the basement membrane or located inside the stromal compartment were counted and divided by total number of pan-cytokeratin positive cells. *, *P* < 0.05 (*t* test, *n* = 3, mean ± SD). Scale bars: 50 μm. **H–M,** Immunostaining of mouse prostates for phospho-Myosin Light Chain 2 (Ser19; phospho-MLC2^Ser19^) to identify levels of cell contractility and Rho-kinase (ROCK) activation in *Pten^fl/fl^Kras^G12V^* cohorts at 100 days (**H–J**) and *Pten^fl/fl^p53^fl/fl^* cohorts at 100 days (**L** and **M**). Relative to *PlxnB1* intact control (**H**), *PLXNB1^W^*^T^ expression lowers MLC2 phosphorylation in the *Pten^fl/fl^Kras^G12V^* cohort (**I**). *PLXNB1^P1597L^* expression increases MLC2 phosphorylation on both backgrounds (**J** and **M**). Scale bars, 100 μm (left image) and 30 μm (right image). **K–N,** H-score quantitation of phospho-MLC2^Ser19^ staining. Epithelial cells were divided into three categories according to staining intensity (strong/medium/low). H-score = 1 × (% «low staining» cells) + 2 × (% «medium staining» cells) + 3 × (% «strong staining» cells). (*n* = 3, 5 fields per sample, *t* test). *, *P* < 0.05 (*t* test, *n* = 3, mean ± SD).

Primary tumors from all cohorts of *Pten^fl/fl^Kras^G12V^* or *Pten^fl/fl^p53^fl/fl^* mice were immunostained for the epithelial marker pan-cytokeratin at the early timepoint of 100 days (before the onset of metastasis) and the percentage of tumors breaking into the stroma was scored (Fig. 5). Overexpression of *PLXNB1^P1597L^*in prostate epithelial cells increased invasion in both *Pten^fl/fl^Kras^G12V^* (Fig. 5A–D) and *Pten^fl/fl^p53^fl/fl^* (Fig. 5E–G) models (2.6%–8%, *P* < 0.05 and 1.6%–2.3%, *P* < 0.05, respectively). In contrast, overexpression of WT Plexin-B1 in prostate epithelial cells of *Pten^fl/fl^Kras^G12V^* mice suppressed tumor cell invasion into the stroma (*P* < 0.05). These results show that mutant Plexin-B1 enhances metastasis and WT Plexin-B1 inhibits metastasis at an early stage in the metastatic process.

### *PLXNB1^P1597L^* Expression Correlates with Rho/ROCK Pathway Activation in Mouse Prostate Tumors

Plexin-B1 activates RhoA and RhoC through PDZRhoGEF/LARG (13), which bind to the C-terminus of Plexin-B1, and inactivates RhoA and RhoC through p190RhoGAP activation. Plexin-B1–mediated activation of RhoA/C is a key pathway promoting metastasis in ErbB2-mouse models of breast cancer (24). To establish whether Plexin-B1 might signal via RhoA/C to promote metastasis in the mouse models of prostate cancer, phosphorylation of myosin light chain (phospho-MLC2^Ser19^—a marker of ROCK activation (44)) was evaluated in tumors of *Pten^fl/fl^Kras^G12V^* and *Pten^fl/fl^p53^fl/fl^* models (Fig. 5).

Overexpression of *PLXNB1^P1597L^* significantly increased MLC2 phosphorylation H-score in both *Pten^fl/fl^Kras^G12V^* (Fig. 5J) and *Pten^fl/fl^p53^fl/fl^* (Fig. 5M) models (4.8-fold and 3.3-fold increase in the percentage of cells with strong staining for *Pten^fl/fl^Kras^G12V^PLXNB1^P1597L^* and *Pten^fl/fl^p53^fl/fl^PLXNB1^P1597L^*, respectively, *P* = <0.05; Fig. 5).

In contrast, overexpression of WT Plexin-B1 in the *Pten^fl/fl^Kras^G12V^* model decreased MLC2 phosphorylation in tumors (*P* = <0.05; Fig. 5I). These results suggest that Plexin-B1 promotes metastasis at least in part, via the Rho-ROCK pathway.

### Inhibition of Rho/ROCK Signaling by Deletion of RhoA/C or PDZRhoGEF Suppresses Metastasis in the *Pten^fl/fl^Kras^G12V^PLXNB1^P1597L^* Model

**FIGURE 6:**
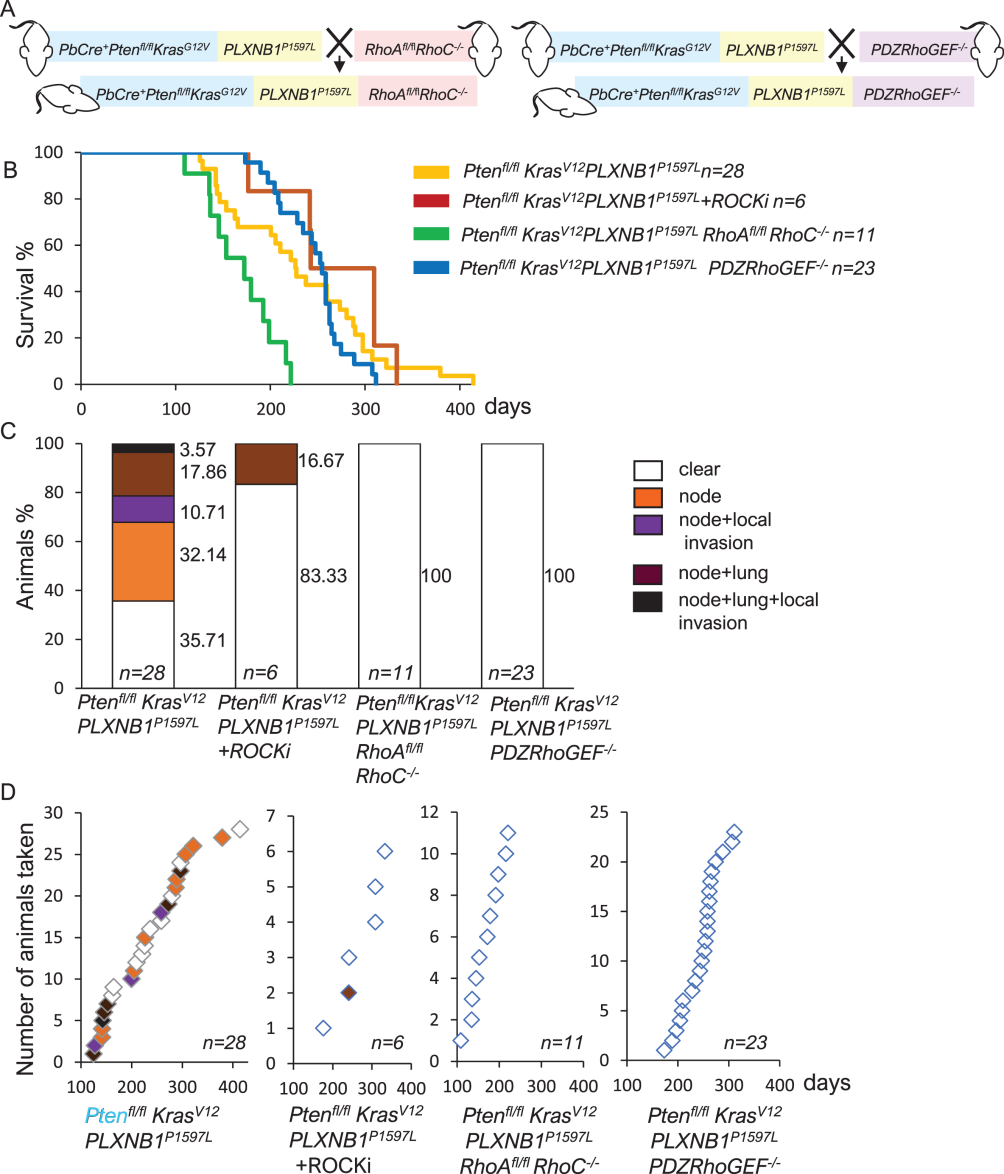
Inhibition of Rho/ROCK signaling by deletion of RhoA/C or PDZRhoGEF suppresses metastasis in the *Pten^fl/fl^Kras^G12V^PLXNB1^P1597L^* model. **A,** Schematic diagram of generation of *Pten^fl/fl^Kras^G12V^PLXNB1^P1597L^RhoA^fl/fl^ RhoC^−/−^* and *Pten^fl/fl^Kras^G12V^PLXNB1^P1597L^ PDZRhoGEF^−/−^* mice. **B,** Kaplan–Meier survival curves for untreated *Pten^fl/fl^Kras^G12V^PLXNB1^P1597L^* mice (*n* = 28), mice treated 1 mg/kg of ROCK inhibitor GSK269962 (*n* = 6) or two cohorts with Rho/ROCK pathway genetic deletions: *Pten^fl/fl^Kras^G12V^PLXNB1^P1597L^ RhoA^fl/fl^ RhoC^−/−^* (*n* = 11) and *Pten^fl/fl^Kras^G12V^PLXNB1^P1597L^ PDZRhoGEF^−/−^* (*n* = 23) mice. Primary prostate tumor growth was the major reason for euthanasia. Cohorts with either ROCK inhibitor treatment (median survival 275.5 days, log-rank test; *z* = 1, *P* = 0.32) or *PDZRhoGEF* deletion (median survival 254 days, log-rank test; *z* = 0.49, *P* = 0.63) had no significant changes in animal survival compared with untreated *Pten^fl/fl^Kras^G12V^PLXNB1^P1597L^* cohort (median survival 226.5 days). *Pten^fl/fl^Kras^G12V^PLXNB1^P1597L^ RhoA^fl/fl^ RhoC^−/−^* showed decrease in survival due to penis skin wart growth (log-rank test; *z* = 3.24, *P* = 0.00119). **C,** Percentages of animals affected/not affected by metastasis in *Pten^fl/fl^Kras^G12V^PLXNB1^P1597L^* cohorts. Following necropsy, mice were categorized according to their metastatic outcome: no metastatic deposits (white), lymph node metastasis (orange), lymph node metastasis combined with invasion into peritoneum or pelvic muscle (purple), combined lymph node and lung metastasis (brown), animals with both lymph node and lung metastasis combined with invasion into peritoneum or pelvic muscle (black). **D,** Timing and type of metastatic deposits in *Pten^fl/fl^Kras^G12V^PLXNB1^P1597L^* cohorts.

We next investigated whether the increase in metastasis observed upon expression of *PLXNB1^P1597L^* in *Pten^fl/fl^Kras^G12V^* mice was dependent on RhoA/C expression. *Pten^fl/fl^Kras^G12V^* mice were crossed with mice containing a conditional inactivation of RhoA (*RhoA^fl/^*^fl^*)* combined with constitutive deletion of RhoC (*RhoC^−/−^;*Fig. 6A)*. Pten^fl/fl^Kras^G12V^PLXNB1^P1597L^ RhoA^fl/^*^fl^*RhoC^−/−^* mice had a marked reduction in life span (Fig. 6B) largely attributed to enhanced skin wart formation around the penis area. Importantly, deletion of RhoA/C in *Pten^fl/fl^Kras^G12V^PLXNB1^P1597L^* mice resulted in complete elimination of metastases (Fig. 6C and D).

Plexin-B1 activates RhoA/C by activation of PDZRhoGEF, which binds to the C-terminal of Plexin-B1 (13). To assess the contribution of PDZRhoGEF /Rho/ROCK signaling to metastatic tumor progression in *Pten^fl/fl^Kras^G12V^PLXNB1^P1597L^* mice, we crossed these mice with mice that harbor deletion of PDZ RhoGEF (*PDZRhoGEF^−/−^;*Fig. 6A). Survival was not affected in the *PDZRhoGEF^−/−^* cohort (Fig. 6B). However, deletion of PDZRhoGEF completely inhibited metastasis (Fig. 6C and D).

Consistent with these results, treatment of *Pten^fl/fl^Kras^G12V^PLXNB1^P1597L^* mice with ROCK inhibitor GSK269962 showed similar survival data to the untreated group (Fig. 6B) but had a significant decrease in metastases (*χ*^2^ test, *P* = 0.033; Fig. 6C and D).

These results indicate that the increase in metastasis observed upon *PLXNB1^P1597L^* expression in *Pten^fl/fl^Kras^G12V^* mice is dependent on RhoA/C signaling

### Germline Deletion of *PlxnB1* Suppresses Invasion and Metastasis in *Pten^fl/fl^Kras^G12V^* and *Pten^fl/fl^p53^fl/fl^* Mice

**FIGURE 7:**
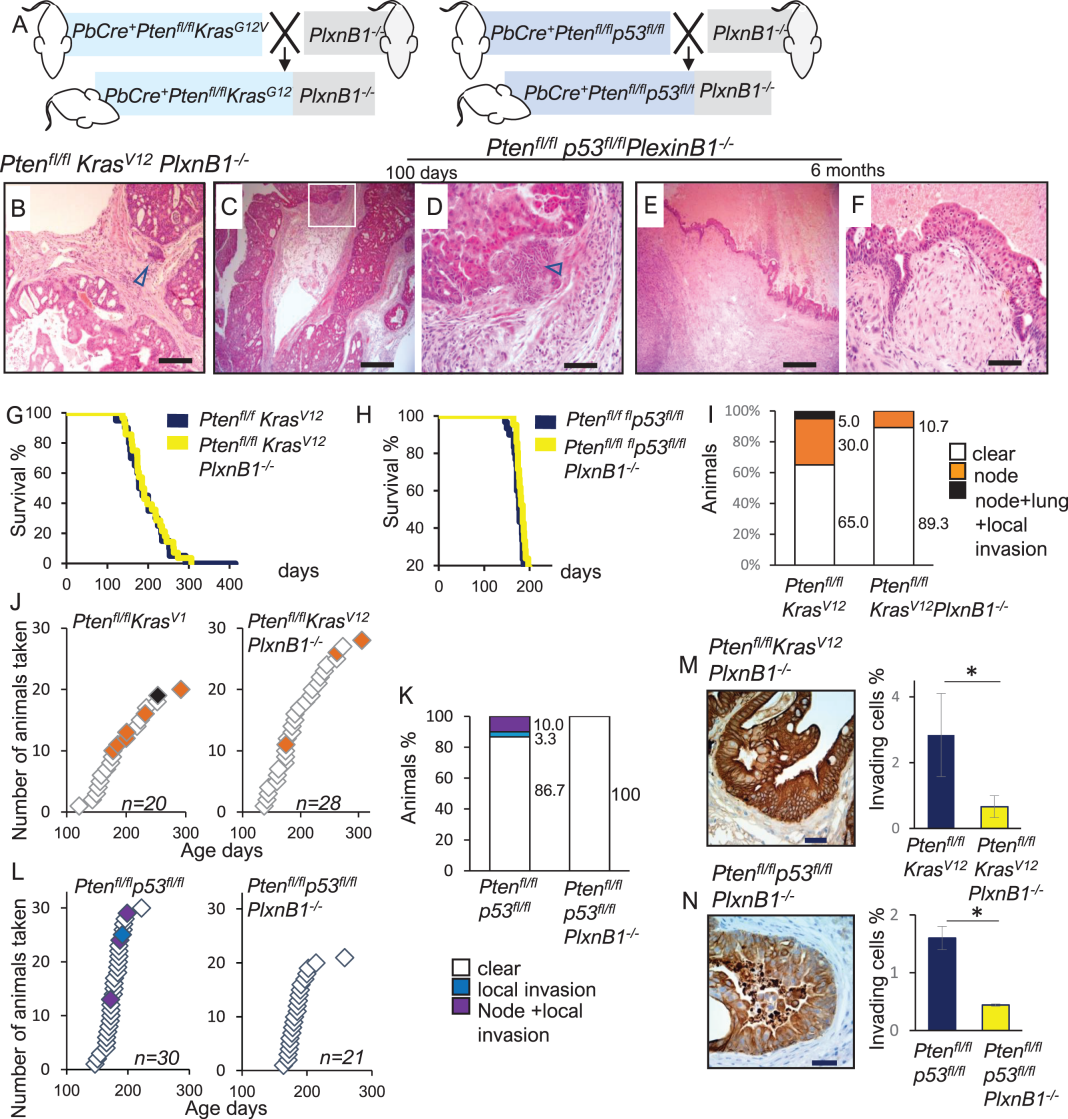
Germline deletion of PlexinB1 decreases metastasis in *Pten^fl/fl^Kras^G12V^* and *Pten^fl/fl^p53^fl/fl^* mice. **A,** Schematic diagram of generation of *Pten^fl/fl^Kras^G12V^PlxnB1^−/−^* and *Pten^fl/fl^p53^fl/fl^PlxnB1^−/−^* mice. **B,** H&E staining of *Pten^fl/fl^Kras^G12V^PlxnB1^−/−^* prostate (scale bar: 200 μm). **C** and **D**, Invasive adenocarcinoma in prostates of *Pten^fl/fl^p53^fl/fl^PlxnB1^−/−^* mice at day 100 timepoint showing sarcomatoid deposits next to epithelium (marked with arrow), scale bar: 500 μm (**C**), 100 μm (**D**). Sarcomatoid tumors from prostates of 6-month-old *Pten^fl/fl^p53^fl/fl^PlxnB1^−/−^* mice. Scale bar: 500 μm (**E**), 50 μm (**F**). **G,** Kaplan–Meier survival curves for *Pten^fl/fl^Kras^G12V^* (*n* = 20), and *Pten^fl/fl^Kras^G12V^PlxnB1^−/−^* (*n* = 28), cohorts. Primary prostate tumor growth was the major reason for euthanasia. **H,** Kaplan–Meier survival curves for *Pten^fl/fl^p53^fl/fl^* (*n* = 30) and *Pten^fl/fl^p53^fl/fl^PlxnB1^−/−^* (*n* = 21) cohorts. **I** and **J,***PlxnB1* germline deletion suppress metastasis in *Pten^fl/fl^Kras^G12V^* mouse model of prostate cancer. Mice were categorized according to their metastatic outcome: no metastatic deposits (white), lymph node metastasis (orange), both lymph node and lung metastasis combined with invasion into peritoneum or pelvic muscle (black). **I,** Percentages of animals with metastases in *Pten^fl/fl^Kras^G12V^* cohorts. **J,** Timing and type of metastatic deposits in *Pten^fl/fl^Kras^G12V^* (*n* = 20), and *Pten^fl/fl^Kras^G12V^ PlxnB1^−/−^* mice (*n* = 28). **K** and **L**, *PlxnB1* germline deletion suppress metastasis in *Pten^fl/fl^p53^fl/fl^* mouse model of prostate cancer. **K,** Percentage of mice with metastases in *Pten^fl/fl^p53^fl/fl^* cohorts: no metastatic deposits (white), invasion into peritoneum or pelvic muscle (blue), lymph node metastasis combined with invasion into peritoneum or pelvic muscle (purple). **L**, Timing and type of metastatic deposits in *Pten^fl/fl^p53^fl/fl^* (*n* = 30) and *Pten^fl/fl^p53^fl/fl^PlxnB1^−/−^* (*n* = 21) mice. See Table 1 for statistical analyses. **M** and **N,***PlxnB1* deletion decreases local invasion by prostate tumor cells. Immunostaining of prostates of 100-day-old mice with cytokeratin AE1/AE3 (pan-cytokeratin) to identify prostate epithelial cells breaking basement membrane and invading stroma in *Pten^fl/fl^Kras^G12V^* (**M**) and *Pten^fl/fl^p53^fl/fl^PlxnB1^−/−^* (**N**) mice. Scale bars, 50 μm. Graphs show quantitation of invasion in the *Pten^fl/fl^Kras^G12V^* and *Pten^fl/fl^p53^fl/fl^* backgrounds—Pan-cytokeratin positive cells breaking the basement membrane or located inside the stromal compartment were counted and divided by total number of pan-cytokeratin positive cells. *, *P* < 0.05 (*t* test, *n* = 3, mean ± SD).

We have shown that prostate-specific overexpression of mutant (*P1597L)* Plexin-B1 promotes metastasis, while prostate-specific WT Plexin-B1 had the opposite effect. Endogenous Plexin-B1 is expressed by a variety of cell types including endothelial cells and Plexin-B1 activation promotes angiogenesis (45). To model the effect of deletion of Plexin-B1 in all cells, we next crossed both *Pten^fl/fl^Kras^G12V^* and *Pten^fl/fl^p53^fl/fl^* lines with mice containing a germline deletion of Plexin-B1 (Fig. 7A). As before, one cohort was established for euthanasia at a fixed timepoint of 100 days and one for euthanasia when required for welfare reasons*.* Plexin-B1 expression was absent from all tissues in *PlxnB1^−/−^* mice, as expected (Supplementary Fig. S2B) and no overt differences in histology of primary tumors were observed, compared with the respective parental lines (Fig. 7B–F).

*PlxnB1* ablation made no significant difference to the survival of either *Pten^fl/fl^Kras^G12V^* or *Pten^fl/fl^p53^fl/fl^* mice (Fig. 7G and H). Median survival of *Pten^fl/fl^Kras^G12V^PlxnB1^−/−^* mice was 187.5 days, compared with 182 days for *Pten^fl/fl^Kras^G12V^* mice; median survival of *Pten^fl/fl^p53^fl/fl^* mice was 185 days compared with 177 days for the *Pten^fl/fl^p53^fl/fl^* parental line. Consistent with these findings, germline deletion of Plexin-B1 had a negligible effect on cell proliferation in either model at 100 days, as demonstrated by *Ki67* staining of prostate epithelial cells (Supplementary Fig. S9A–C).

Germline deletion of Plexin-B1 reduced metastasis substantially in both *Pten^fl/fl^Kras^G12V^* (Fig. 7I and J) and *Pten^fl/fl^p53^fl/fl^* (Fig. 7K and L) models (*P* = 0.0411 for *Pten^fl/fl^Kras^G12V^* mice; Table 1). Deletion of Plexin-B1 resulted in a 3-fold reduction in the number of mice with metastases in the *Pten^fl/fl^Kras^G12V^* model: 35% of *Pten^fl/fl^Kras^G12V^* mice had node metastases, including one mouse with an additional lung metastasis; in contrast, 10.7% of *Pten^fl/fl^Kras^G12V^PlxnB1^−/−^* mice had node metastases and no distant metastases were found (Fig. 7I and J; Supplementary Fig. S8). Deletion of *PlxnB1* in *Pten^fl/fl^p53^fl/fl^* mice completely blocked local invasion and metastases (Fig. 7K and L).

Consistent with these results, deletion of Plexin-B1 significantly reduced invasion into the stroma in both *Pten^fl/fl^Kras^G12V^* (2.6%–0.7%, *P* < 0.05; Fig. 7M) and *Pten^fl/fl^p53^fl/fl^* (1.6%–0.4%, *P* < 0.05; Fig. 7N) backgrounds, showing that PlexinBI is required for the earlier stages of metastasis in these models.

Deletion of Plexin-B1 reduced semiquantitative MLC2 phosphorylation H-score, with a 2-fold reduction in cells with “strong” staining in both models upon Plexin-B1 ablation in 100-day-old mice (*P* = <0.05; Supplementary Fig. S9D and S9E), suggesting that deletion of Plexin-B1 reduced Rho/ROCK signaling in these tumors.

Together, these results indicate that systemic inhibition of Plexin-B1 has potential as a treatment for prostate cancer.

## Discussion

Our results show that Plexin-B1 status has a major effect on prostate cancer metastasis (see Supplementary Fig. S10 for summary). Overexpression of WT Plexin-B1 targeted specifically to the prostate epithelial cells of *Pten^fl/fl^Kras^G12V^* mice, decreased invasion and metastasis in comparison with *Pten^fl/fl^Kras^G12V^* mice expressing normal levels of Plexin-B1. Sema4D, the ligand for Plexin-B1, is expressed by cells in the prostate stroma and tumor-associated macrophages (46) and Sema4D secreted from the tumor microenvironment may act as a repellent cue to inhibit migration and invasion of tumor cells expressing WT Plexin-B1, confining the tumor cells to the primary tumor mass. Sema4D produced by tumor cells may also act as an autocrine or paracrine signal to suppress migration; nonpolarized activation of Plexin-B1 over the whole cell results in cell collapse *in vitro* (47). Alternatively, ligand-independent Plexin-B1 signaling due to WT Plexin-B1 overexpression and receptor clustering (19), may repress migration and invasion. B-type plexins have been shown to act in a ligand-independent manner to suppress the rate of cell division through the detection of mechanical forces, in embryonic skin development (48).

In direct contrast to overexpression of WT Plexin-B1, similar levels of mutant (*P1597L*) Plexin-B1 in the mouse models significantly increased metastasis.

These findings reflect our previous results where overexpression of WT Plexin-B1 decreased migration and invasive capacity of HEK293 cells and overexpression of the *P1597L* mutant form of Plexin-B1 increased motility and invasion (26). The increase in invasion and metastasis of prostate cancers observed in the *Pten^fl/fl^Kras^G12V^PLXNB1^P1597L^* and *Pten^fl/fl^p53^fl/fl^PLXNB1^P1597L^* mice may result from a change in response of tumor cells to semaphorins produced by the stroma—a switch from repulsion to attraction (4). Sema4D has been shown to promote or suppress migration and invasion depending on cellular context and the Plexin-B1 coreceptors expressed by the responding cell (5). The contrasting results from similar levels of overexpression of the WT and mutant proteins argue against the findings being an artefact of overexpression *per se*.

The signaling mechanism by which the single clinically relevant *Pro1597Leu* amino-acid change converts Plexin-B1 from a metastasis-suppressor to a metastasis-promoter in a *Pten^fl/fl^Kras^G12V^* background, in which the Ras-MAPK pathway is overactivated, is unclear.

Expression of *PLXNB1^WT^* or *PLXNB1^P1597L^* in *Pten^fl/fl^Kras^G12V^* decreased or increased MLC2 phosphorylation, respectively, while deletion of RhoA/C or PDZRhoGEF in *Pten^fl/fl^Kras^G12V^PLXNB1^P1597L^* mice inhibited metastasis. Deletion of RhoA/C in *Pten^fl/fl^Kras^G12V^PLXNB1^P1597L^* mice shortened their survival. This reduction in survival may have contributed to the reduction in metastasis observed in these mice. However, deletion of PDZRhoGEF, a guanine nucleotide exchange factor for RhoA, had little effect on survival, yet it significantly reduced metastasis, showing that the PDZRhoGEF-Rho-ROCK pathway is required for metastasis in *Pten^fl/fl^Kras^G12V^PLXNB1^P1597L^* mice. Together, these results implicate Rho signaling in *PLXNB1^P1597L^-*induced metastasis. RhoA/C are upregulated in many cancer types including prostate cancer and promote metastasis in mouse models (49).

The *P1597L* mutation is in the GAP domain of Plexin-B1 and so may disrupt the RapGAP activity of Plexin-B1. Rap has diverse functions in tumor progression (50) and Rap1 activation promotes prostate cancer metastasis (51). Activation of the RapGAP activity of Plexin-B1 (WT) through Rnd1 binding has been shown to inhibit Rap and Ras—Rap1 inhibition leads to derepression of p120 Ras-GAP resulting in Ras inactivation (52). Consequently, WT Plexin-B1 activation inhibits tumor progression in mouse models of metastasis (52). Inactivation of the GAP activity of Plexin-B1 by the *P1597L* mutation is therefore expected to result in Rap1 and Ras activation, promoting metastasis (Supplementary Fig. S10).

The mice models expressing *PLXNB1^P1597L^* showed suppression of primary tumor proliferation and consequently extended survival, yet a considerable increase in metastasis. These findings support a model in which expression of mutant Plexin-B1 switched prostate tumor cells from a proliferative to an invasive phenotype. This led to longer survival of the mice, as there were fewer local complications due to primary tumor mass, but increased tumor cell motility, escape from the primary tumor site and metastasis to lymph nodes and organs. It is important to note that although suffering from morbidity due to local advancement if not treated, men with prostate cancer rarely, if ever, die of complications of the primary tumor (unlike with mice), but rather of the metastatic burden.

Survival times can bias the detectable rates of metastasis. However, there was no correlation between the age of *Pten^fl/fl^Kras^G12V^PLXNB1^P1597L^* mice and metastases even though survival was increased in this cohort. Furthermore, deletion of *PDZRhoGEF* in *Pten^fl/fl^Kras^G12V^PLXNB1^P1597L^* mice did not significantly affect their survival but did reduce metastasis, showing that the increase in metastasis in *Pten^fl/fl^Kras^G12V^PLXNB1^P1597L^* mice cannot be explained by an increase in survival. Expression of *PLXNB1^WT^* in *Pten^fl/fl^Kras^G12V^* mice or knockout of *PlxnB1* in both models did not affect survival but did significantly reduce metastasis.

While overexpression of *PLXNB1^P1597L^* increased invasion and metastasis in both *Pten^fl/fl^Kras^G12V^* and *Pten^fl/fl^p53^fl/fl^* models, a marked difference in histology and means of spread were seen between tumors from the different backgrounds. Expression of *PLXNB1^P1597L^* in *Pten^fl/fl^Kras^G12V^* mice resulted in tumors with an epithelial phenotype and a significant increase in metastasis to lymph nodes and lung, metastasis in this background occurring via the lymphatic and/or via blood vessel route. In contrast, expression of mutant Plexin-B1 in *Pten^fl/fl^p53^fl/fl^* mice resulted in an increase in tumors with a mesenchymal phenotype which were predominantly locally invasive into surrounding tissues such as the peritoneum, pelvic or bladder muscle, or vas deferens. Consistent with these results, Sema3C drives epithelial–mesenchymal transition in prostate cells (53) promoting a spindle-like morphology. These results demonstrate the context dependence of specific semaphorin/plexin-mediated signaling pathways controlling metastasis.

Most human metastatic prostate cancers exhibit AR-dependent epithelial histology. One limitation of the models used in this study is the finding that *Pten^fl/fl^Kras^G12V^PLXNB1^P1597L^* mice and especially *Pten^fl/fl^p53^fl/fl^PLXNB1^P1597^* mice develop metastases with predominantly sarcomatoid and squamous histology. This may have implications on the clinical significance of these models.

While the *Pten^fl/fl^Kras^G12V^* and *Pten^fl/fl^p53^fl/fl^* models used in this study recapitulate alterations in the *P13K, Ras/Raf/MEK/ERK,* and *p53* signaling pathways (32), the effect of mutation of *PLXNB1* in a background of other commonly found changes in human prostate cancer, such as AR mutation or amplification, was not assessed here.

Plexin-B1 is overexpressed in some cancers and appears to act as a tumor suppressor gene in others (54). Indeed, high levels of Plexin-B1 expression predict longer overall survival in bladder carcinoma, head and neck squamous cell carcinoma, and kidney papillary renal cell carcinoma but shorter overall survival in thymoma and kidney renal clear-cell carcinoma (data from Kaplan-Meier Plotter Pan-Cancer Tool; https://kmplot.com/analysis/index.php?p=back ground; ref. 55). Data on the prognostic significance of Plexin-B1 in prostate cancer are complicated by the use of different baseline comparators. A large-scale gene expression comparison between prostate cancer and normal tissue (32) showed Plexin-B1 expression was altered in 30% of patients with prostate cancer (z-score = ±2) and Plexin-B1 expression downregulation was three times more common (22.67%) in prostate cancer than its increase (7.33%). However, other large-scale genomics projects using a different baseline (diploid tumor samples instead of normal prostate tissue) have suggested that Plexin-B1 upregulation is more common than its decrease [cBioportal (56, 57) summarized in Supplementary Table S3]. Notably, cBioportal data also show that Plexin-B1 expression is inversely correlated with PTEN (Spearman correlation −0.374, *P* = 1.34E-05, *q*-value = 2.56E-05). It is interesting to speculate that elevated levels of WT Plexin-B1 may suppress metastasis in a *PTEN*-deleted tumor, but if the Plexin-B1 acquires a mutation it may switch to a driver of aggressive metastatic disease.

Germline deletion of Plexin-B1 in *Pten^fl/fl^Kras^G12V^* and *Pten^fl/fl^p53^fl/fl^* mice significantly decreased metastasis in comparison with *Pten^fl/fl^Kras^G12V^* and *Pten^fl/fl^p53^fl/fl^* mice with normal levels and patterns of Plexin-B1 expression, demonstrating that Plexin-B1 is required for metastasis in these mouse models. Consistent with these results, whole body knockout of Plexin-B1 inhibited metastasis in ErbB2-expressing models of breast cancer (24). Plexin-B1 is expressed by endothelial cells (45, 46) in the tumor microenvironment in addition to tumor epithelial cells. Activation of Plexin-B1 on endothelial cells by Sema4D which is expressed by tumor cells (58), promotes angiogenesis (45, 46, 58); therefore, whole-body knockout of Plexin-B1 in our mouse models would inhibit Sema4D-induced angiogenesis and this may contribute to the decrease in metastasis observed.

Normal levels of WT Plexin-B1 in tumor cells may promote metastasis while overexpression of *PLXNB1^WT^* reduces metastasis. This hypothesis is consistent with our *in vitro* findings: knockdown of Plexin-B1 reduces migration and invasion in prostate cancer cells expressing ErbB2, while activation of endogenous Plexin-B1 with Sema4D promotes migration and invasion. In contrast, forced overexpression of *PLXNB1^WT^* decreases invasion and migration, while overexpression of *PLXNB1^P1597L^* has the opposite effect in transfected cells (59). Overexpression of *PLXNB1^WT^* may alter the balance of Rho activation (via PDZRhoGEF/LARG) and Rap/Ras Rho inhibition (via the RapGAP activity of Plexin-B1; Supplementary Fig. S11). *PLXNB1^WT^* overexpression may also result in the activation of negative feedback loops.

Human prostate tumors are highly heterogenous and consist of a complex mixture of clones of different genetic make-up which complicates analysis (60). The use of mouse models of a defined genetic background allows the effect of the many different genetic changes found in human tumors to be analyzed separately. Current treatments for metastatic prostate cancer are effective only in the short term, highlighting the need for new therapies for late-stage disease. To test such therapies, preclinical models in which metastasis is driven by clinically relevant mutations, such as those we have developed here, are a key requirement. Together, our results have demonstrated that Plexin-B1 has a complex yet significant role in metastasis and is a potential therapeutic target to block the lethal spread of prostate cancer.

## Supplementary Material

Figure SF1 Generation of PLXNB1WT and PLXNB1P1597L mice

Figure SF2 Expression of PlexinB1 in mouse prostate and tumours

Figure SF3 Metastases in Ptenfl/flKrasG12V mice

Figure SF4 Metastatic deposits in Ptenfl/flKrasG12VPLXNB1P1597L mice

Figure SF5 Metastatic deposit in Ptenfl/flKrasG12VPLXNB1WT mouse

Figure SF6 Metastatic deposits in Ptenfl/flp53fl/fl mice

Figure SF7 Metastatic deposits in Ptenfl/flp53fl/flPLXNB1P1597L mice

Figure SF8 Metastatic deposits in Ptenfl/flKrasG12VPlxnB1-/- mice

Figure SF9 Proliferation and ROCK activation in Ptenfl/flKrasG12V PlxnB1-/- and Ptenfl/flp53fl/fl PlxnB1-/- mice

Figure SF10 Summary of results

Figure SF11 Model of possible effects of P1597L mutation

Table ST1 Summary of age of mice and weight of primary tumours

Table ST2 List of antibodies used

Table ST3 Expression of Plexin-B1 in human prostate cancer
