# Supplementary material for: Plexin-B1 Mutation Drives Metastasis in Prostate Cancer Mouse Models
Source: Cancer Res Commun. 2023 Mar 16;3(3):444–58. doi: 10.1158/2767-9764.CRC-22-0480 (PMC10019359; doi:10.1158/2767-9764.CRC-22-0480)
Supplement: Figure SF1 — Generation of PLXNB1WT and PLXNB1P1597L mice [file crc-22-0480-s01.pptx]

## Slide 1
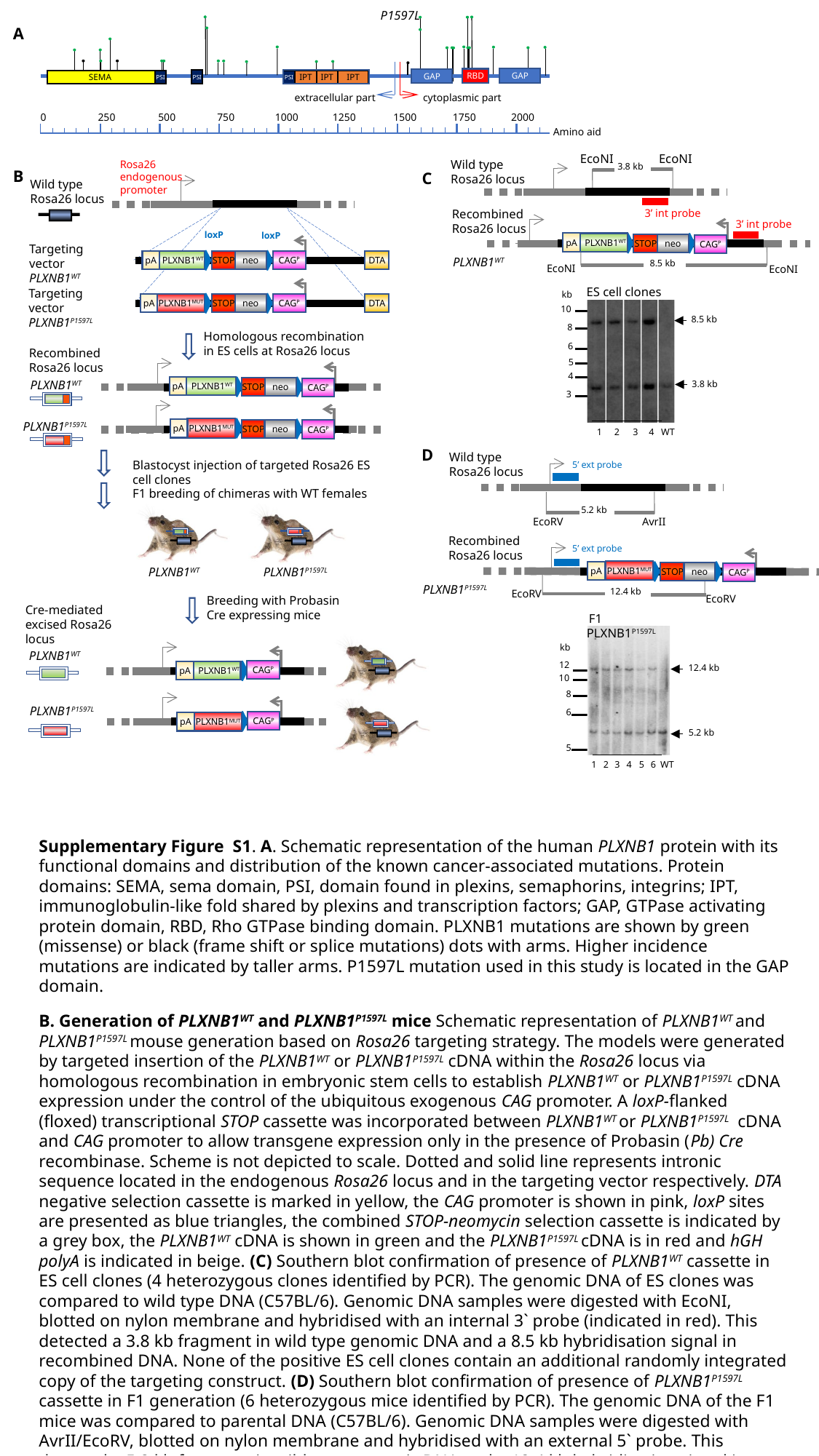

P1597L
A
RBD
GAP
GAP
PSI
SEMA
PSI
PSI
IPT
IPT
IPT
cytoplasmic part
extracellular part
0
250
500
750
1000
1250
1500
1750
2000
Amino aid
 EcoNI
 EcoNI
Wild type Rosa26 locus
Rosa26 endogenous promoter
 3.8 kb
B
C
Wild type Rosa26 locus
Recombined Rosa26 locus
 3’ int probe
 3’ int probe
STOP
neo
loxP
loxP
pA
PLXNB1WT
CAGP
Targeting vector PLXNB1WT
pA
PLXNB1WT
STOP
neo
CAGP
DTA
 PLXNB1WT
 8.5 kb
 EcoNI
 EcoNI
ES cell clones
kb
10
8.5 kb
8
6
5
4
3.8 kb
3
 1
 2
 3
 4
 WT
Targeting vector PLXNB1P1597L
pA
PLXNB1MUT
STOP
neo
CAGP
DTA
Homologous recombination in ES cells at Rosa26 locus
Recombined Rosa26 locus
 PLXNB1WT
pA
PLXNB1WT
STOP
neo
CAGP
 PLXNB1P1597L
PLXNB1MUT
pA
neo
STOP
CAGP
D
Wild type Rosa26 locus
Blastocyst injection of targeted Rosa26 ES cell clones
F1 breeding of chimeras with WT females
 5’ ext probe
 5.2 kb
 EcoRV
AvrII
 5’ ext probe
STOP
neo
PLXNB1MUT
pA
CAGP
 12.4 kb
 EcoRV
 EcoRV
Recombined Rosa26 locus
 PLXNB1WT
 PLXNB1P1597L
 PLXNB1P1597L
Breeding with Probasin Cre expressing mice
Cre-mediated excised Rosa26 locus
 F1 PLXNB1P1597L
kb
12.4 kb
6
5.2 kb
5
12
10
8
1
2
3
4
5
6
WT
 PLXNB1WT
CAGP
pA
PLXNB1WT
PLXNB1P1597L
CAGP
pA
PLXNB1MUT
Supplementary Figure S1. A. Schematic representation of the human PLXNB1 protein with its functional domains and distribution of the known cancer-associated mutations. Protein domains: SEMA, sema domain, PSI, domain found in plexins, semaphorins, integrins; IPT, immunoglobulin-like fold shared by plexins and transcription factors; GAP, GTPase activating protein domain, RBD, Rho GTPase binding domain. PLXNB1 mutations are shown by green (missense) or black (frame shift or splice mutations) dots with arms. Higher incidence mutations are indicated by taller arms. P1597L mutation used in this study is located in the GAP domain.
B. Generation of PLXNB1WT and PLXNB1P1597L mice Schematic representation of PLXNB1WT and PLXNB1P1597L mouse generation based on Rosa26 targeting strategy. The models were generated by targeted insertion of the PLXNB1WT or PLXNB1P1597L cDNA within the Rosa26 locus via homologous recombination in embryonic stem cells to establish PLXNB1WT or PLXNB1P1597L cDNA expression under the control of the ubiquitous exogenous CAG promoter. A loxP-flanked (floxed) transcriptional STOP cassette was incorporated between PLXNB1WT or PLXNB1P1597L cDNA and CAG promoter to allow transgene expression only in the presence of Probasin (Pb) Cre recombinase. Scheme is not depicted to scale. Dotted and solid line represents intronic sequence located in the endogenous Rosa26 locus and in the targeting vector respectively. DTA negative selection cassette is marked in yellow, the CAG promoter is shown in pink, loxP sites are presented as blue triangles, the combined STOP-neomycin selection cassette is indicated by a grey box, the PLXNB1WT cDNA is shown in green and the PLXNB1P1597L cDNA is in red and hGH polyA is indicated in beige. (C) Southern blot confirmation of presence of PLXNB1WT cassette in ES cell clones (4 heterozygous clones identified by PCR). The genomic DNA of ES clones was compared to wild type DNA (C57BL/6). Genomic DNA samples were digested with EcoNI, blotted on nylon membrane and hybridised with an internal 3` probe (indicated in red). This detected a 3.8 kb fragment in wild type genomic DNA and a 8.5 kb hybridisation signal in recombined DNA. None of the positive ES cell clones contain an additional randomly integrated copy of the targeting construct. (D) Southern blot confirmation of presence of PLXNB1P1597L cassette in F1 generation (6 heterozygous mice identified by PCR). The genomic DNA of the F1 mice was compared to parental DNA (C57BL/6). Genomic DNA samples were digested with AvrII/EcoRV, blotted on nylon membrane and hybridised with an external 5` probe. This detected a 5.2 kb fragment in wild type genomic DNA and a 12.4 kb hybridisation signal in recombined DNA.
