# Supplementary material for: Plexin-B1 Mutation Drives Metastasis in Prostate Cancer Mouse Models
Source: Cancer Res Commun. 2023 Mar 16;3(3):444–58. doi: 10.1158/2767-9764.CRC-22-0480 (PMC10019359; doi:10.1158/2767-9764.CRC-22-0480)
Supplement: Figure SF2 — Expression of PlexinB1 in mouse prostate and tumours [file crc-22-0480-s02.pptx]

## Slide 1
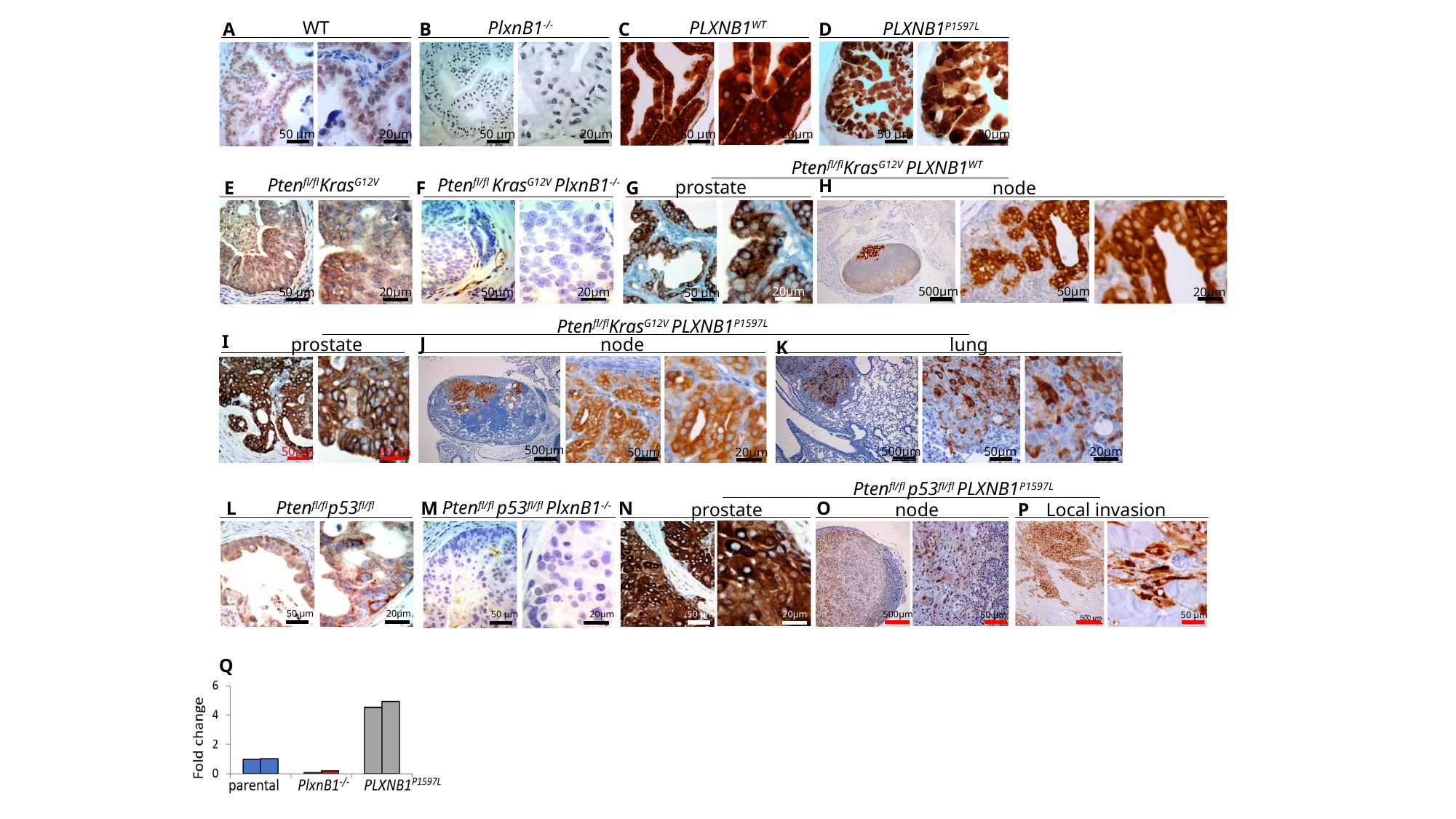

WT
PlxnB1-/-
PLXNB1WT
PLXNB1P1597L
A
B
C
D
50 μm
20μm
50 μm
20μm
50 μm
20μm
50 μm
20μm
Ptenfl/flKrasG12V PLXNB1WT
Ptenfl/flKrasG12V
Ptenfl/fl KrasG12V PlxnB1-/-
H
prostate
node
 E
F
G
500μm
50μm
20μm
50 μm
20μm
50μm
20μm
50 μm
20μm
Ptenfl/flKrasG12V PLXNB1P1597L
I
J
prostate
node
lung
K
500μm
20μm
50μm
50μm
20μm
500μm
50μm
20μm
Ptenfl/fl p53fl/fl PLXNB1P1597L
Ptenfl/flp53fl/fl
Ptenfl/fl p53fl/fl PlxnB1-/-
L
M
 N
 O
prostate
node
Local invasion
P
50 μm
20μm
50 μm
20μm
50 μm
20μm
500μm
50 μm
50 μm
Q

## Slide 2
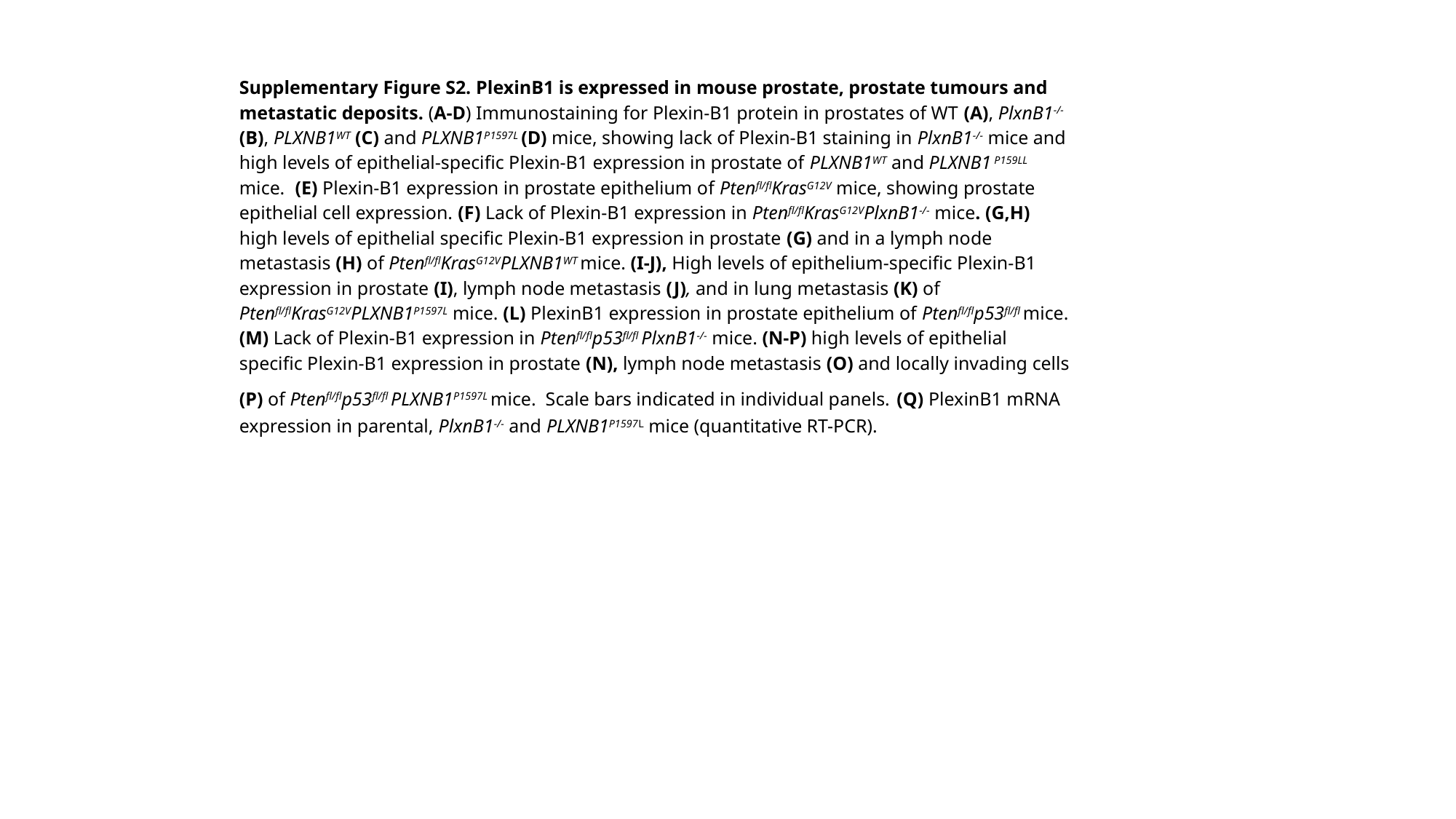

Supplementary Figure S2. PlexinB1 is expressed in mouse prostate, prostate tumours and metastatic deposits. (A-D) Immunostaining for Plexin-B1 protein in prostates of WT (A), PlxnB1-/- (B), PLXNB1WT (C) and PLXNB1P1597L (D) mice, showing lack of Plexin-B1 staining in PlxnB1-/- mice and high levels of epithelial-specific Plexin-B1 expression in prostate of PLXNB1WT and PLXNB1 P159LL mice. (E) Plexin-B1 expression in prostate epithelium of Ptenfl/flKrasG12V mice, showing prostate epithelial cell expression. (F) Lack of Plexin-B1 expression in Ptenfl/flKrasG12VPlxnB1-/- mice. (G,H) high levels of epithelial specific Plexin-B1 expression in prostate (G) and in a lymph node metastasis (H) of Ptenfl/flKrasG12VPLXNB1WT mice. (I-J), High levels of epithelium-specific Plexin-B1 expression in prostate (I), lymph node metastasis (J), and in lung metastasis (K) of Ptenfl/flKrasG12VPLXNB1P1597L mice. (L) PlexinB1 expression in prostate epithelium of Ptenfl/flp53fl/fl mice. (M) Lack of Plexin-B1 expression in Ptenfl/flp53fl/fl PlxnB1-/- mice. (N-P) high levels of epithelial specific Plexin-B1 expression in prostate (N), lymph node metastasis (O) and locally invading cells (P) of Ptenfl/flp53fl/fl PLXNB1P1597L mice. Scale bars indicated in individual panels. (Q) PlexinB1 mRNA expression in parental, PlxnB1-/- and PLXNB1P1597L mice (quantitative RT-PCR).
