# Supplementary material for: Plexin-B1 Mutation Drives Metastasis in Prostate Cancer Mouse Models
Source: Cancer Res Commun. 2023 Mar 16;3(3):444–58. doi: 10.1158/2767-9764.CRC-22-0480 (PMC10019359; doi:10.1158/2767-9764.CRC-22-0480)
Supplement: Figure SF3 — Metastases in Ptenfl/flKrasG12V mice [file crc-22-0480-s03.pdf]

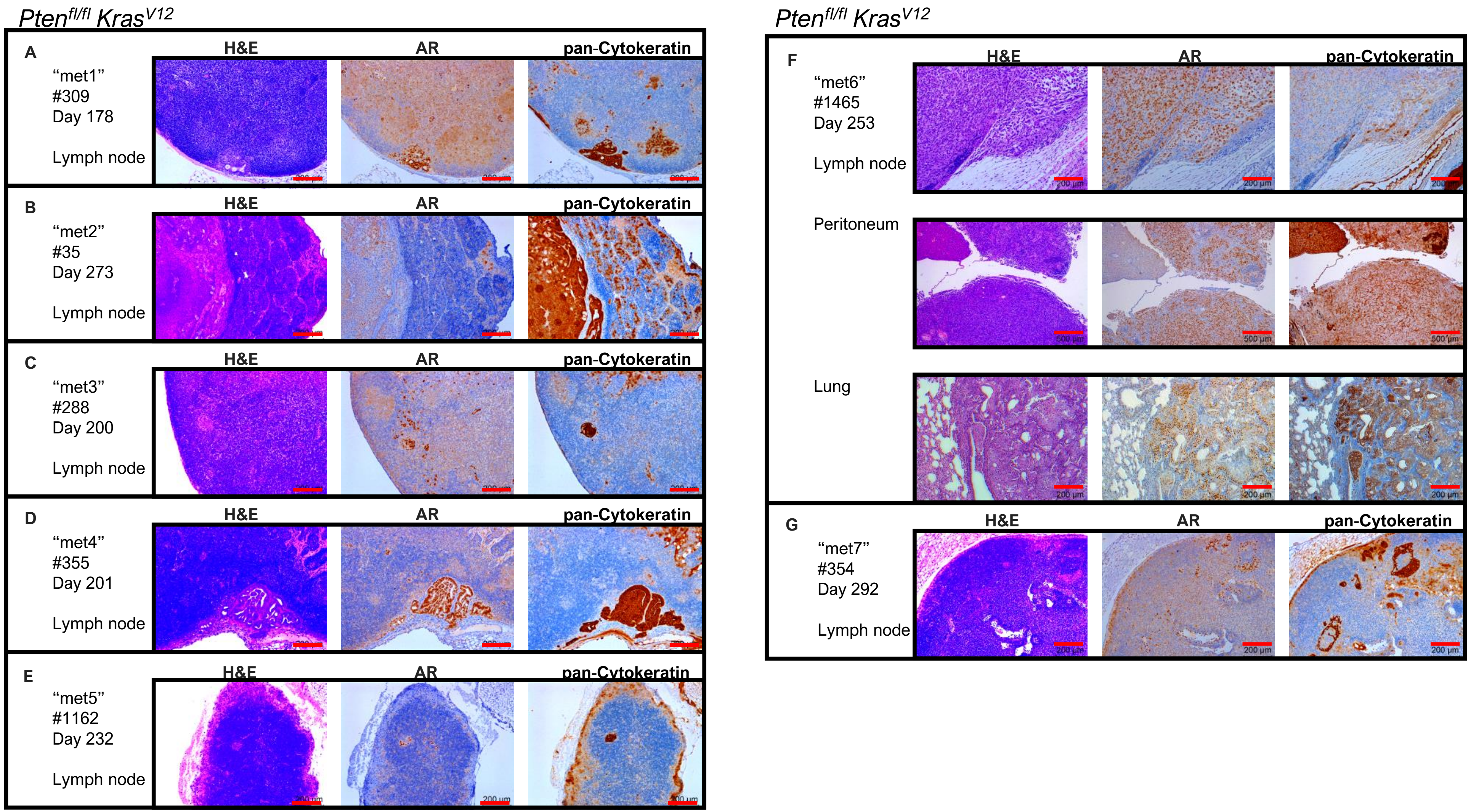

Supplementary Figure 3. Metastatic deposits in *Pten$^{fl/fl}$Kras^{G12V}* mouse cohort stained for H&E, androgen receptor (AR) and pan-cytokeratin. Metastatic deposits were observed in 7 *Pten$^{fl/fl}$ Kras^{V12}* cohort animals, met1 (#309, 178 days old, **A**), met2 (#35, 273 days old, **B**), met3 (#288, 200 days old, **C**), met4 (#355, 201 days old, **D**), met5 (#1162, 232 days old, **E**), met6 (#1465, 253 days old, **F**), met7 (#354, 292 days old, **G**). H&E (left image), AR (middle image) and pan-cytokeratin (right image). Scale bars are 200µm (apart from **F**, peritoneum, 500µm).
