# Supplementary material for: Plexin-B1 Mutation Drives Metastasis in Prostate Cancer Mouse Models
Source: Cancer Res Commun. 2023 Mar 16;3(3):444–58. doi: 10.1158/2767-9764.CRC-22-0480 (PMC10019359; doi:10.1158/2767-9764.CRC-22-0480)
Supplement: Figure SF5 — Metastatic deposit in Ptenfl/flKrasG12VPLXNB1WT mouse [file crc-22-0480-s05.pptx]

## Slide 1
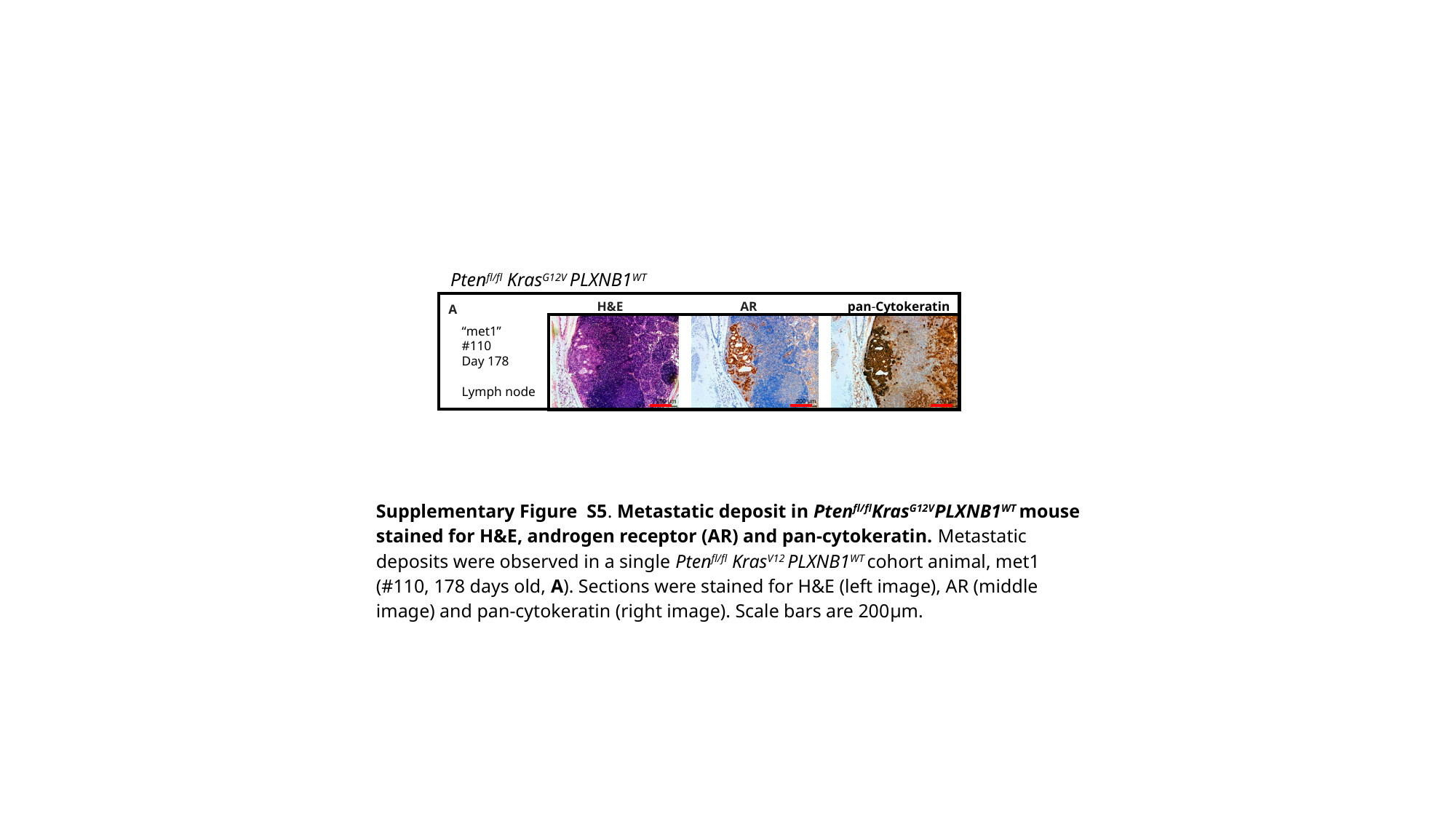

Ptenfl/fl KrasG12V PLXNB1WT
H&E
AR
pan-Cytokeratin
A
“met1”
#110
Day 178
Lymph node
Supplementary Figure S5. Metastatic deposit in Ptenfl/flKrasG12VPLXNB1WT mouse stained for H&E, androgen receptor (AR) and pan-cytokeratin. Metastatic deposits were observed in a single Ptenfl/fl KrasV12 PLXNB1WT cohort animal, met1 (#110, 178 days old, A). Sections were stained for H&E (left image), AR (middle image) and pan-cytokeratin (right image). Scale bars are 200μm.
