# Supplementary material for: Plexin-B1 Mutation Drives Metastasis in Prostate Cancer Mouse Models
Source: Cancer Res Commun. 2023 Mar 16;3(3):444–58. doi: 10.1158/2767-9764.CRC-22-0480 (PMC10019359; doi:10.1158/2767-9764.CRC-22-0480)
Supplement: Figure SF6 — Metastatic deposits in Ptenfl/flp53fl/fl mice [file crc-22-0480-s06.pptx]

## Slide 1
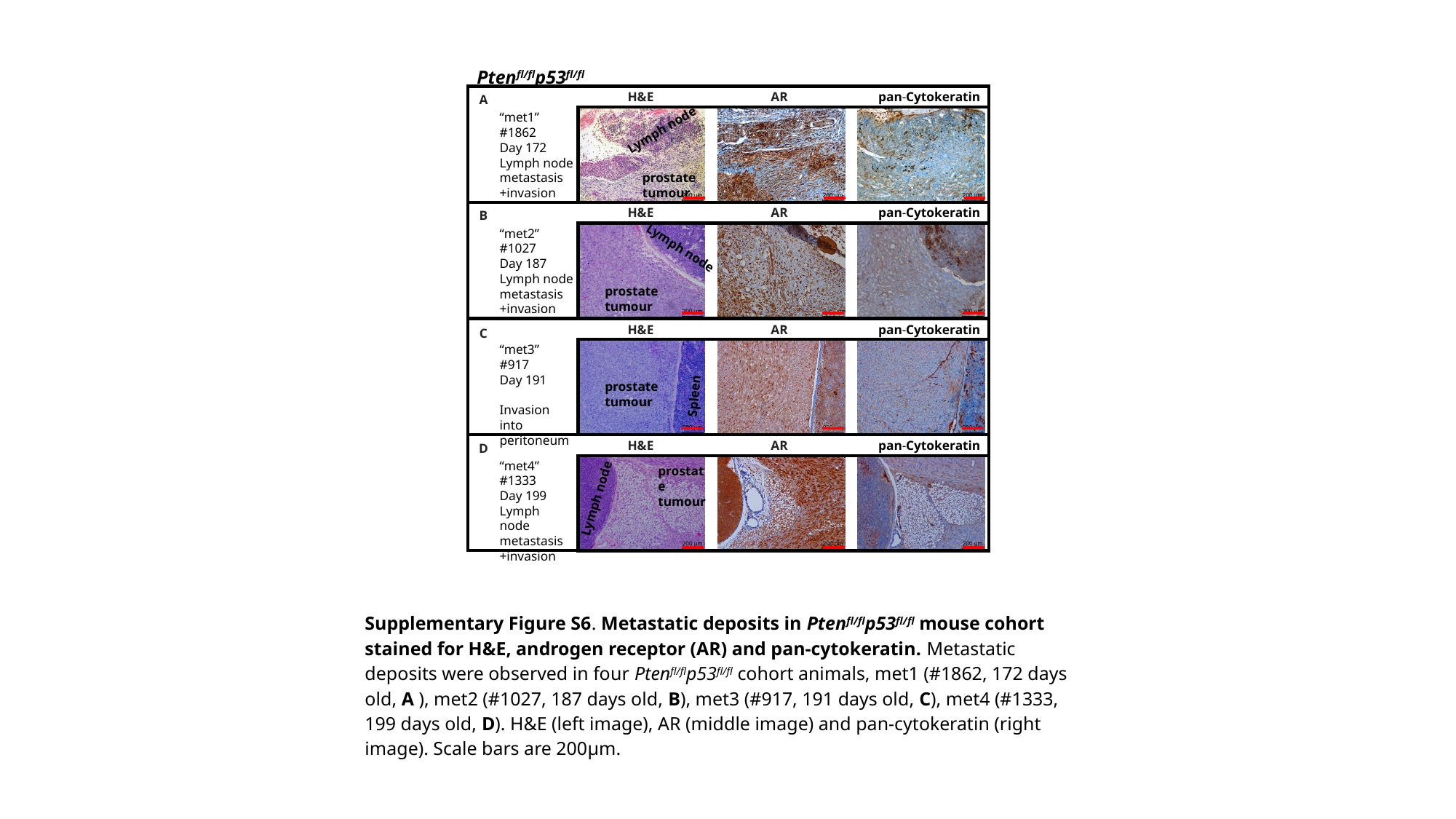

Ptenfl/flp53fl/fl
H&E
AR
pan-Cytokeratin
A
“met1”
#1862
Day 172
Lymph node metastasis +invasion
Lymph node
prostate tumour
H&E
AR
pan-Cytokeratin
B
“met2”
#1027
Day 187
Lymph node metastasis +invasion
Lymph node
prostate tumour
H&E
AR
pan-Cytokeratin
C
“met3”
#917
Day 191
Invasion into peritoneum
prostate tumour
Spleen
H&E
AR
pan-Cytokeratin
D
“met4”
#1333
Day 199
Lymph node metastasis +invasion
prostate tumour
Lymph node
Supplementary Figure S6. Metastatic deposits in Ptenfl/flp53fl/fl mouse cohort stained for H&E, androgen receptor (AR) and pan-cytokeratin. Metastatic deposits were observed in four Ptenfl/flp53fl/fl cohort animals, met1 (#1862, 172 days old, A ), met2 (#1027, 187 days old, B), met3 (#917, 191 days old, C), met4 (#1333, 199 days old, D). H&E (left image), AR (middle image) and pan-cytokeratin (right image). Scale bars are 200μm.
