# Supplementary material for: Plexin-B1 Mutation Drives Metastasis in Prostate Cancer Mouse Models
Source: Cancer Res Commun. 2023 Mar 16;3(3):444–58. doi: 10.1158/2767-9764.CRC-22-0480 (PMC10019359; doi:10.1158/2767-9764.CRC-22-0480)
Supplement: Figure SF7 — Metastatic deposits in Ptenfl/flp53fl/flPLXNB1P1597L mice [file crc-22-0480-s07.pdf]

9

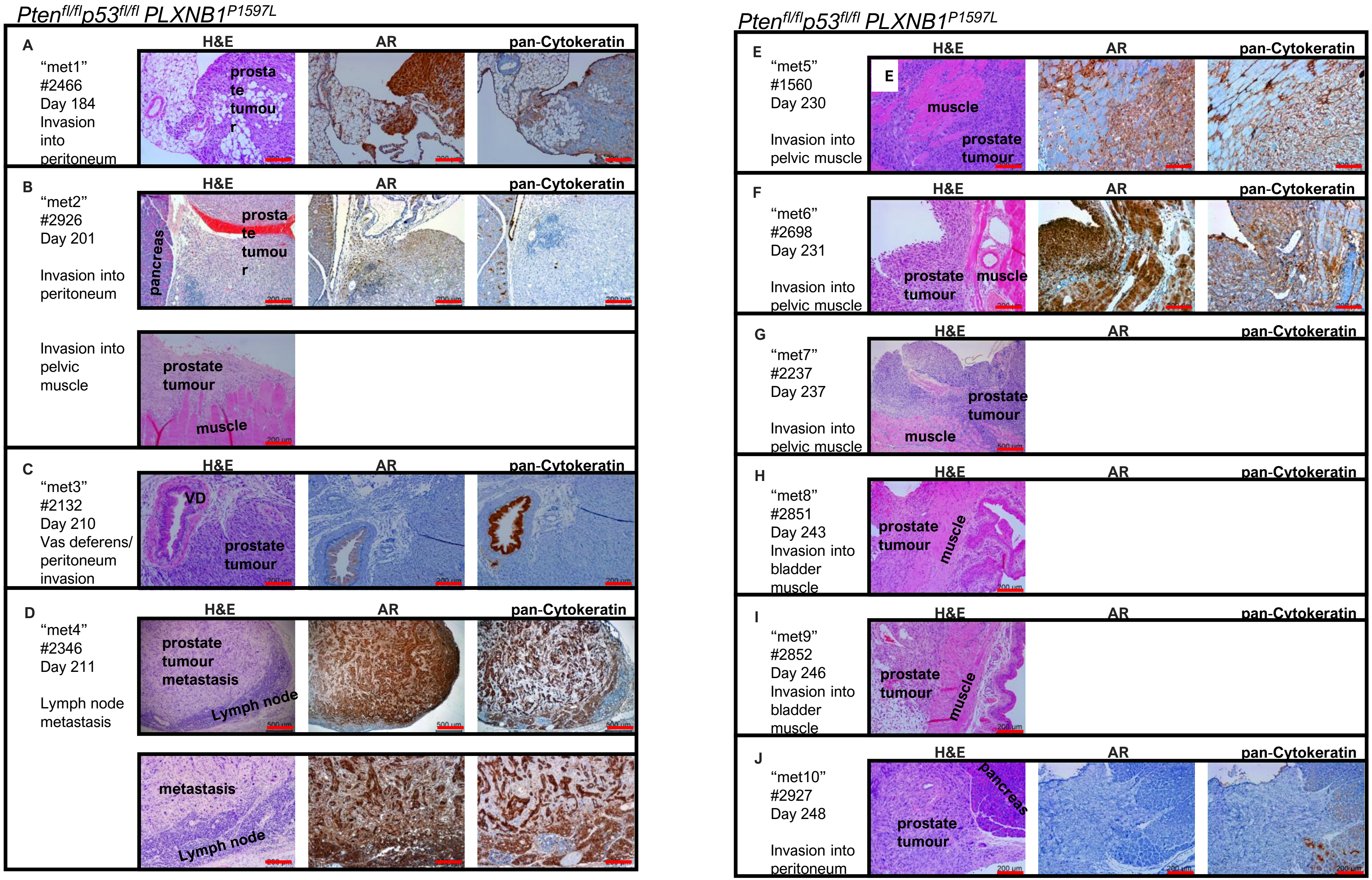
Pten fl/fl p53 fl/fl PLXNB1 P1597L
A
"met1"
#2466
Day 184
Invasion into peritoneum
H&E
AR
pan-Cytokeratin
prostate tumour
B
"met2"
#2926
Day 201
Invasion into peritoneum
pancreas
prostate tumour
Invasion into pelvic muscle
muscle
C
"met3"
#2132
Day 210
Vas deferens/ peritoneum invasion
VD
D
"met4"
#2346
Day 211
Lymph node metastasis
prostate tumour metastasis
Lymph node
metastasis
Pten fl/fl p53 fl/fl PLXNB1 P1597L
E
"met5"
#1560
Day 230
Invasion into pelvic muscle
F
"met6"
#2698
Day 231
G
"met7"
#2237
Day 237
H
"met8"
#2851
Day 243
Invasion into bladder muscle
I
"met9"
#2852
Day 246
J
"met10"
#2927
Day 248
pancreas
200 µm
500 µm

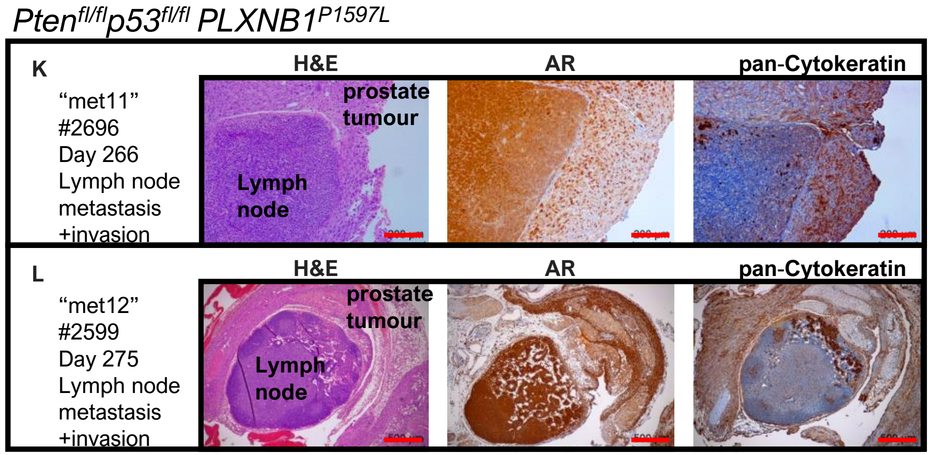

Supplementary Figure 7. Metastatic deposits in *Pten$^{fl/fl}$p53$^{fl/fl}$PLXNB1^{P1597L}* mouse cohort stained for H&E, androgen receptor (AR) and pan-cytokeratin. Metastatic deposits were observed in twelve *Pten$^{fl/fl}$p53$^{fl/fl}$ PLXNB1^{P1597L}* cohort animals, met1 (#2466, 172 days old, **A**), met2 (#2926, 201 days old, **B**), met3 (#2132, 210 days old, **C**), met4 (#2346, 211 days old, **D**) met5 (#1560, 230 days old, **E**) met6 (#2698, 231 days old, **F**) met7 (#2237, 237 days old, **G**) met8 (#2851, 243 days old, **H**) met9 (#2852, 246 days old, **I**) met10 (#2927, 248 days old, **J**) met11 (#2696, 266 days old, **K**) met12 (#2599, 275 days old, **L**). H&E (left image), AR (middle image) and pan-cytokeratin (right image). Scale bars are 200μm apart from **D** (upper row), **G** and **L** (500 μm)
