# Supplementary material for: Plexin-B1 Mutation Drives Metastasis in Prostate Cancer Mouse Models
Source: Cancer Res Commun. 2023 Mar 16;3(3):444–58. doi: 10.1158/2767-9764.CRC-22-0480 (PMC10019359; doi:10.1158/2767-9764.CRC-22-0480)
Supplement: Figure SF8 — Metastatic deposits in Ptenfl/flKrasG12VPlxnB1-/- mice [file crc-22-0480-s08.pptx]

## Slide 1
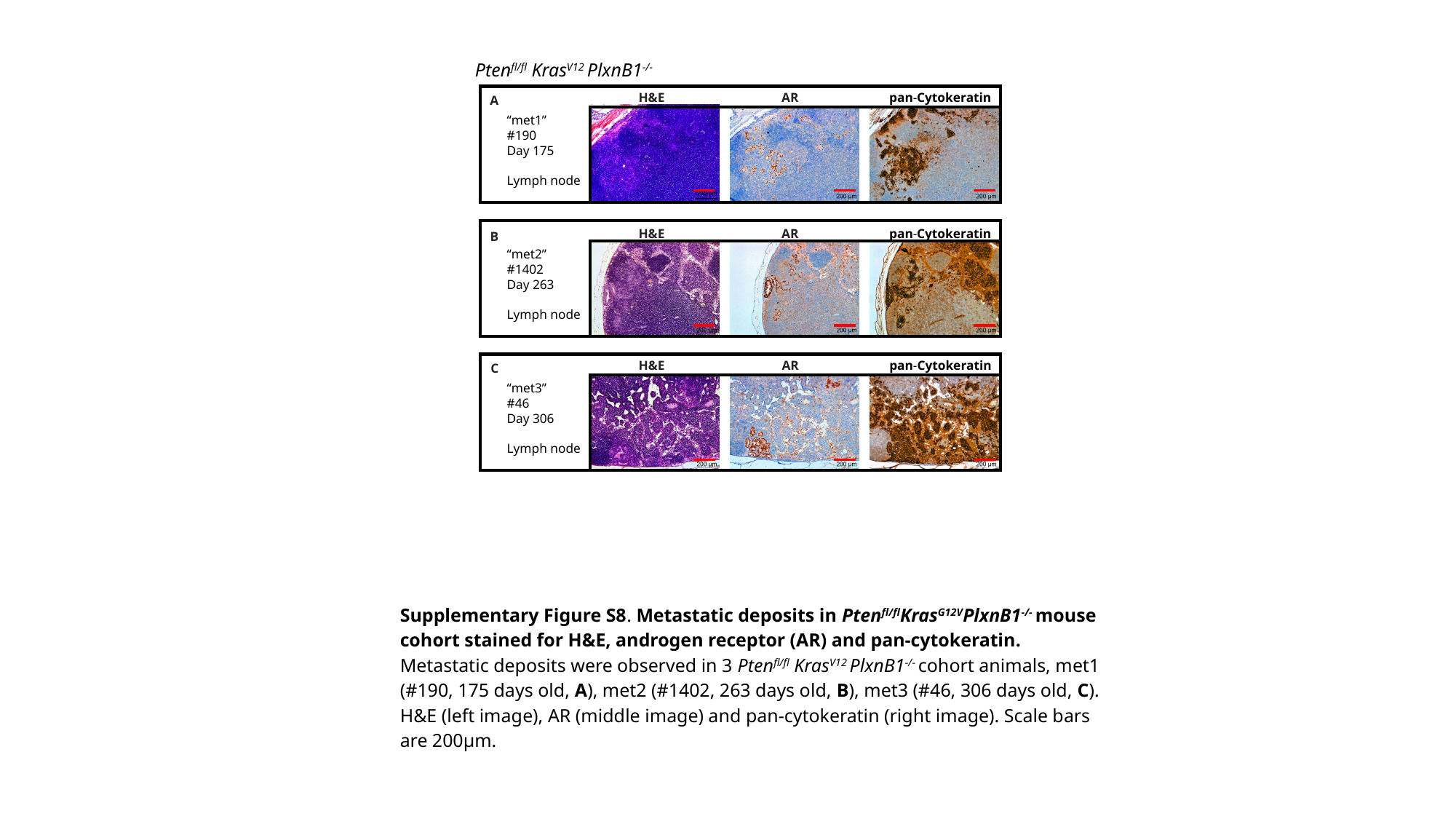

Ptenfl/fl KrasV12 PlxnB1-/-
H&E
AR
pan-Cytokeratin
A
“met1”
#190
Day 175
Lymph node
H&E
AR
pan-Cytokeratin
B
“met2”
#1402
Day 263
Lymph node
H&E
AR
pan-Cytokeratin
C
“met3”
#46
Day 306
Lymph node
Supplementary Figure S8. Metastatic deposits in Ptenfl/flKrasG12VPlxnB1-/- mouse cohort stained for H&E, androgen receptor (AR) and pan-cytokeratin. Metastatic deposits were observed in 3 Ptenfl/fl KrasV12 PlxnB1-/- cohort animals, met1 (#190, 175 days old, A), met2 (#1402, 263 days old, B), met3 (#46, 306 days old, C). H&E (left image), AR (middle image) and pan-cytokeratin (right image). Scale bars are 200μm.
