# Supplementary material for: Plexin-B1 Mutation Drives Metastasis in Prostate Cancer Mouse Models
Source: Cancer Res Commun. 2023 Mar 16;3(3):444–58. doi: 10.1158/2767-9764.CRC-22-0480 (PMC10019359; doi:10.1158/2767-9764.CRC-22-0480)
Supplement: Figure SF9 — Proliferation and ROCK activation in Ptenfl/flKrasG12V PlxnB1-/- and Ptenfl/flp53fl/fl PlxnB1-/- mice [file crc-22-0480-s09.pptx]

## Slide 1
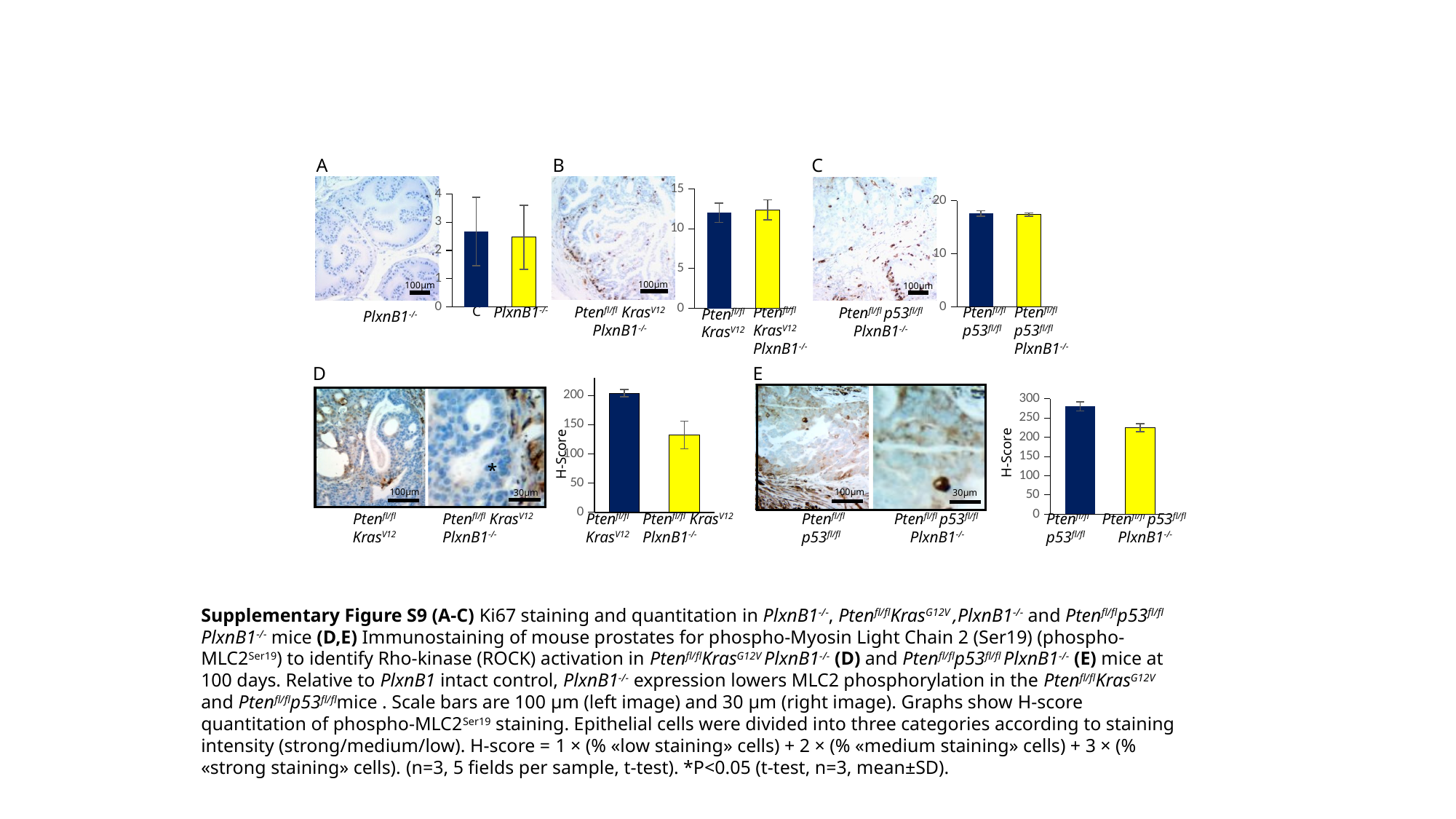

A
B
C
100μm
### Chart
| Category | |
|---|---|Ptenfl/fl KrasV12
100μm
PlxnB1-/-
### Chart
| Category | |
|---|---|
### Chart
| Category | |
|---|---|100μm
C
PlxnB1-/-
Ptenfl/fl KrasV12
PlxnB1-/-
Ptenfl/fl
KrasV12
PlxnB1-/-
Ptenfl/fl p53fl/fl
Ptenfl/fl
p53fl/fl
PlxnB1-/-
Ptenfl/fl p53fl/fl
PlxnB1-/-
D
E
### Chart
| Category | |
|---|---|
100μm
30μm
*
100μm
30μm
### Chart
| Category | |
|---|---|
### Chart
| Category |
|---|H-Score
H-Score
Ptenfl/fl KrasV12
Ptenfl/fl KrasV12 PlxnB1-/-
Ptenfl/fl KrasV12
Ptenfl/fl KrasV12 PlxnB1-/-
Ptenfl/fl p53fl/fl
Ptenfl/fl p53fl/fl
PlxnB1-/-
Ptenfl/fl p53fl/fl
Ptenfl/fl p53fl/fl
PlxnB1-/-
Supplementary Figure S9 (A-C) Ki67 staining and quantitation in PlxnB1-/-, Ptenfl/flKrasG12V ,PlxnB1-/- and Ptenfl/flp53fl/fl PlxnB1-/- mice (D,E) Immunostaining of mouse prostates for phospho-Myosin Light Chain 2 (Ser19) (phospho-MLC2Ser19) to identify Rho-kinase (ROCK) activation in Ptenfl/flKrasG12V PlxnB1-/- (D) and Ptenfl/flp53fl/fl PlxnB1-/- (E) mice at 100 days. Relative to PlxnB1 intact control, PlxnB1-/- expression lowers MLC2 phosphorylation in the Ptenfl/flKrasG12V and Ptenfl/flp53fl/flmice . Scale bars are 100 μm (left image) and 30 μm (right image). Graphs show H-score quantitation of phospho-MLC2Ser19 staining. Epithelial cells were divided into three categories according to staining intensity (strong/medium/low). H-score = 1 × (% «low staining» cells) + 2 × (% «medium staining» cells) + 3 × (% «strong staining» cells). (n=3, 5 fields per sample, t-test). *P<0.05 (t-test, n=3, mean±SD).
