# Supplementary material for: Plexin-B1 Mutation Drives Metastasis in Prostate Cancer Mouse Models
Source: Cancer Res Commun. 2023 Mar 16;3(3):444–58. doi: 10.1158/2767-9764.CRC-22-0480 (PMC10019359; doi:10.1158/2767-9764.CRC-22-0480)
Supplement: Figure SF10 — Summary of results [file crc-22-0480-s10.pptx]

## Slide 1
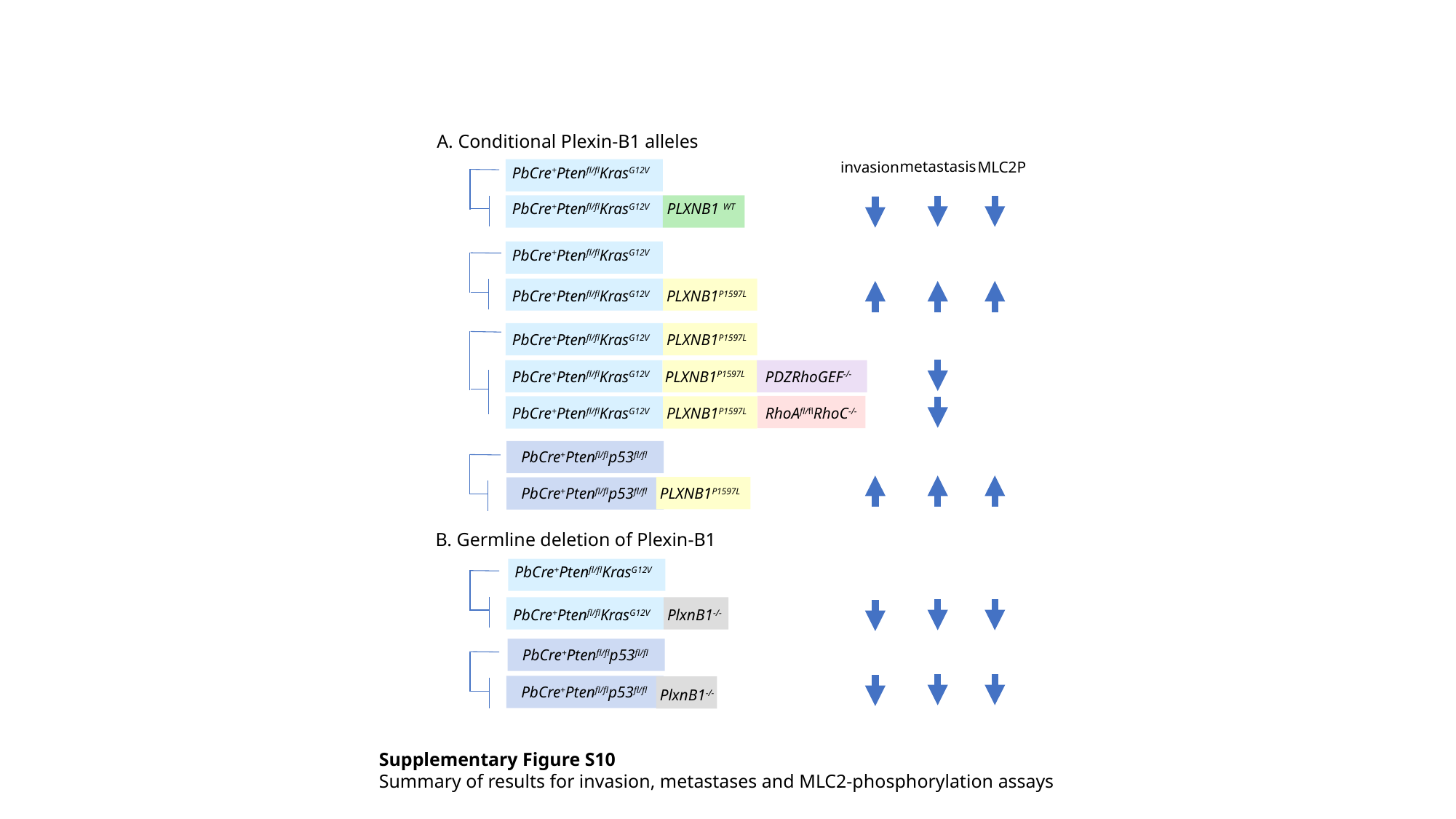

A. Conditional Plexin-B1 alleles
metastasis
invasion
MLC2P
PbCre+Ptenfl/flKrasG12V
PbCre+Ptenfl/flKrasG12V
PLXNB1 WT
PbCre+Ptenfl/flKrasG12V
PbCre+Ptenfl/flKrasG12V
PLXNB1P1597L
PbCre+Ptenfl/flKrasG12V
PLXNB1P1597L
PbCre+Ptenfl/flKrasG12V
PLXNB1P1597L
PDZRhoGEF-/-
RhoAfl/flRhoC-/-
PbCre+Ptenfl/flKrasG12V
PLXNB1P1597L
PbCre+Ptenfl/flp53fl/fl
PLXNB1P1597L
PbCre+Ptenfl/flp53fl/fl
B. Germline deletion of Plexin-B1
PbCre+Ptenfl/flKrasG12V
PbCre+Ptenfl/flKrasG12V
PlxnB1-/-
PbCre+Ptenfl/flp53fl/fl
PbCre+Ptenfl/flp53fl/fl
PlxnB1-/-
Supplementary Figure S10
Summary of results for invasion, metastases and MLC2-phosphorylation assays
