# Supplementary material for: Plexin-B1 Mutation Drives Metastasis in Prostate Cancer Mouse Models
Source: Cancer Res Commun. 2023 Mar 16;3(3):444–58. doi: 10.1158/2767-9764.CRC-22-0480 (PMC10019359; doi:10.1158/2767-9764.CRC-22-0480)
Supplement: Figure SF11 — Model of possible effects of P1597L mutation [file crc-22-0480-s11.pptx]

## Slide 1
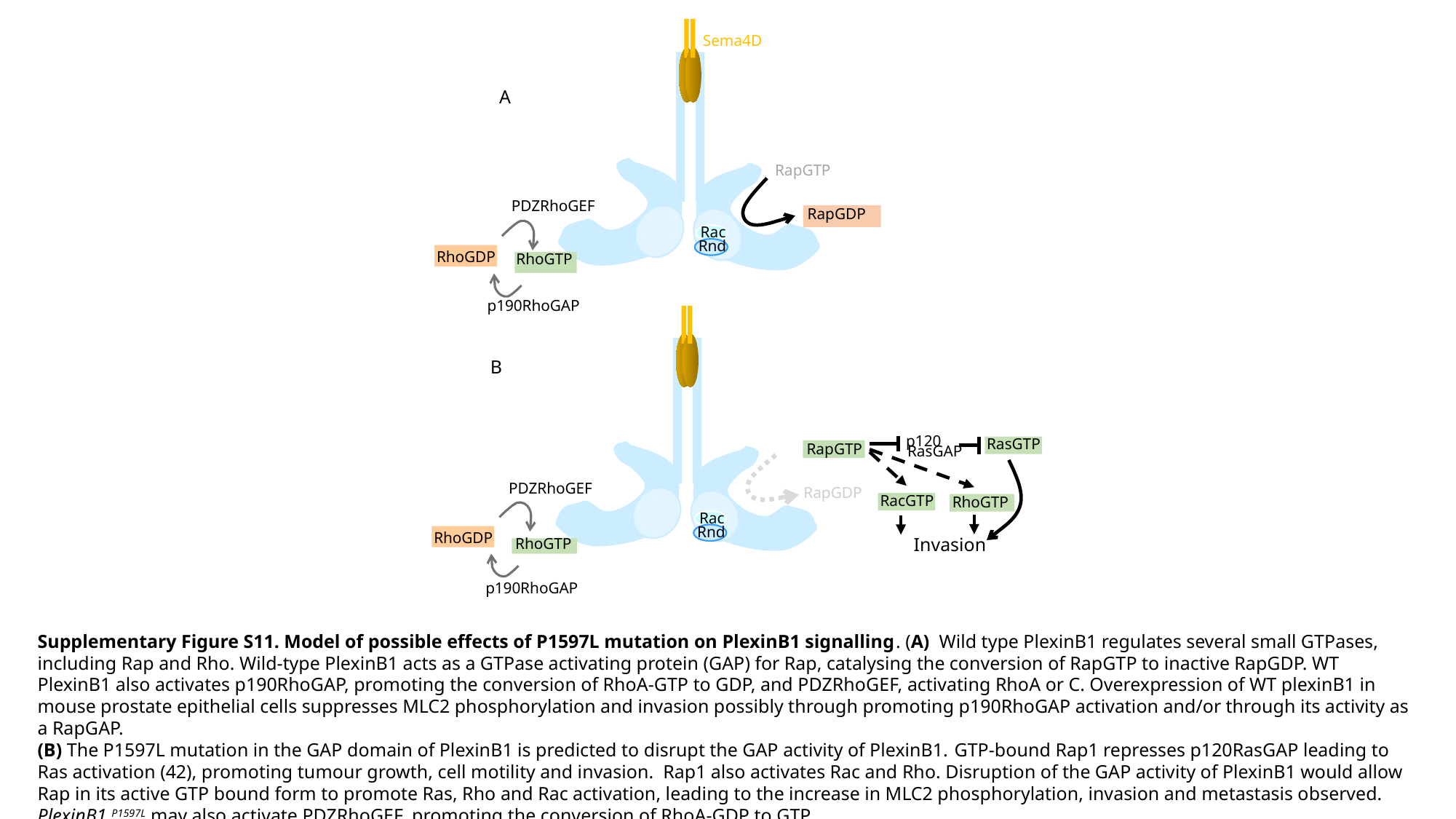

Sema4D
A
RapGTP
PDZRhoGEF
RhoGDP
RhoGTP
p190RhoGAP
RapGDP
Rac
Rnd
B
p120
RasGAP
RasGTP
RapGTP
RacGTP
RhoGTP
PDZRhoGEF
RhoGDP
RhoGTP
p190RhoGAP
RapGDP
Rac
Rnd
Invasion
Supplementary Figure S11. Model of possible effects of P1597L mutation on PlexinB1 signalling. (A) Wild type PlexinB1 regulates several small GTPases, including Rap and Rho. Wild-type PlexinB1 acts as a GTPase activating protein (GAP) for Rap, catalysing the conversion of RapGTP to inactive RapGDP. WT PlexinB1 also activates p190RhoGAP, promoting the conversion of RhoA-GTP to GDP, and PDZRhoGEF, activating RhoA or C. Overexpression of WT plexinB1 in mouse prostate epithelial cells suppresses MLC2 phosphorylation and invasion possibly through promoting p190RhoGAP activation and/or through its activity as a RapGAP.
(B) The P1597L mutation in the GAP domain of PlexinB1 is predicted to disrupt the GAP activity of PlexinB1. GTP-bound Rap1 represses p120RasGAP leading to Ras activation (42), promoting tumour growth, cell motility and invasion. Rap1 also activates Rac and Rho. Disruption of the GAP activity of PlexinB1 would allow Rap in its active GTP bound form to promote Ras, Rho and Rac activation, leading to the increase in MLC2 phosphorylation, invasion and metastasis observed. PlexinB1 P1597L may also activate PDZRhoGEF, promoting the conversion of RhoA-GDP to GTP.
