# Supplementary material for: Plexin-B1 Mutation Drives Metastasis in Prostate Cancer Mouse Models
Source: Cancer Res Commun. 2023 Mar 16;3(3):444–58. doi: 10.1158/2767-9764.CRC-22-0480 (PMC10019359; doi:10.1158/2767-9764.CRC-22-0480)
Supplement: Table ST1 — Summary of age of mice and weight of primary tumours [file crc-22-0480-s12.pdf]

Genotype: *Pten*$^{fl/fl}$ *Kras*^{V12}

	Age	metastatic status	tumour weight (g)
1	121	clear	0.9
2	143	clear	1
3	150	clear	1.5
4	153	clear	2
5	156	clear	1.6
6	158	clear	0.8
7	167	clear	1
8	174	clear	1.1
9	177	clear	0.9
10	178	metastatic node	0.7
11	186	metastatic node	3.6
12	200	metastatic node	1.4
13	201	metastatic node	0.8
14	218	clear	0.8
15	224	clear	1.6
16	232	metastatic node	0.6
17	234	clear	2.2
18	252	clear	2.8
19	253	metastatic node+liver met, lung met	3.9
20	292	metastatic node	2.3

Genotype: *Pten*$^{fl/fl}$ *Kras*^{V12} *PlxnB1*$^{-/-}$

	Age	metastatic status	tumour weight (g)
1	137	clear	0.7
2	142	clear	1.1
3	144	clear	0.9
4	145	clear	0.9
5	159	clear	0.7
6	160	clear	0.8
7	162	clear	0.8
8	173	clear	1
9	174	clear	2.5
10	174	clear	1.7
11	175	metastatic node	1.5
12	177	clear	1.6
13	185	clear	0.7
14	185	clear	1
15	190	clear	1.5
16	190	clear	1.3
17	198	clear	1.9
18	209	clear	1
19	218	clear	1.2
20	220	clear	1.7
21	230	clear	2.2
22	234	clear	3
23	241	clear	2.2
24	245	clear	0.9
25	262	clear	1.3
26	263	metastatic node	1.2
27	273	clear	1.6
28	306	metastatic node	1.4

Genotype: *Pten*$^{fl/fl}$ *Kras*^{V12} *PLXNB1*^{WT}

	Age	metastatic status	tumour weight (g)
1	151	clear	0.7
2	155	clear	0.8
3	166	clear	1.1
4	166	clear	0.8
5	168	clear	1.5
6	170	clear	1.2
7	171	clear	0.8
8	175	clear	1
9	177	clear	2.3
10	178	metastatic node	0.7
11	186	clear	1.5
12	190	clear	1.2
13	195	clear	2.2
14	203	clear	1.4
15	204	clear	0.6
16	206	clear	1.5
17	209	clear	0.9
18	209	clear	1.1
19	210	clear	1.4
20	212	clear	1.6
21	249	clear	0.8
22	250	clear	2.4

Genotype: *Pten*$^{fl/fl}$ *Kras*^{V12} *PLXNB1*^{P1597L}

	Age	metastatic status	tumour weight (g)
1	125	metastatic node+lung met	0.7
2	128	metastatic node+perit met	3.8
3	142	metastatic node	2.5
4	142	metastatic node	1
5	143	metastatic node+lung and perit met	3
6	146	metastatic node+lung met	2
7	153	metastatic node+lung met	1.9
8	162	clear	1.2
9	165	clear	1.3
10	200	metastatic node+liv met	3.5
11	205	metastatic node	1.2
12	210	clear	1.4
13	221	clear	1.6
14	226	clear	0.9
15	227	metastatic node	0.9
16	237	clear	0.9
17	259	clear	1
18	259	metastatic node+perit met	3.5
19	273	metastatic node+lung met	3.5
20	280	clear	0.8
21	287	metastatic node	1.5
22	289	metastatic node	3.1
23	297	metastatic node+lung met	2
24	297	clear	0.8
25	307	metastatic node	5.3
26	322	metastatic node	1.5
27	379	metastatic node	1.4
28	414	clear	3

Genotype: *Pten*$^{fl/fl}$ *p53*$^{fl/fl}$

	Age	metastatic status	tumour weight (g)
1	146	clear	1.8
2	150	clear	2.5
3	155	clear	2.3
4	164	clear	1.9
5	165	clear	4.5
6	165	clear	2
7	167	clear	1.9
8	169	clear	2.5
9	171	clear	3.7
10	172	clear	5
11	172	clear	2.4
12	172	clear	2.5
13	172	Lymph node metastasis +invasion	2.8
14	176	clear	4
15	177	clear	4.5
16	177	clear	3.8
17	178	clear	3.3
18	183	clear	3.9
19	183	clear	4.7
20	184	clear	2.9
21	185	clear	3.7
22	185	clear	2.4
23	185	clear	4.1
24	187	Lymph node metastasis +invasion	2.9
25	191	Invasion into peritoneum, sarc next to spleen and pancreas	3.8
26	192	clear	4
27	193	clear	2.4
28	195	clear	2.4
29	199	Lymph node metastasis +invasion	4
30	222	clear	3.9

Genotype: *Pten*$^{fl/fl}$ *p53*$^{fl/fl}$ *PlxnB1*$^{-/-}$

	Age	metastatic status	tumour weight (g)
1	164	clear	4.4
2	171	clear	4.8
3	171	clear	4.1
4	173	clear	4.5
5	174	clear	6.9
6	174	clear	5.8
7	177	clear	5.2
8	178	clear	4.1
9	180	clear	5.2
10	181	clear	6
11	185	clear	3.2
12	185	clear	6.4
13	187	clear	5.6
14	188	clear	1.9
15	190	clear	4
16	191	clear	3.2
17	197	clear	5.6
18	200	clear	6
19	202	clear	6.2
20	214	clear	7.1
21	258	clear	5.4

Genotype: *Pten*$^{fl/fl}$ *p53*$^{fl/fl}$ *PLXNB1*^{1597I}

	Age	metastatic status	tumour weight (g)
1	145	clear	2.7
2	159	clear	1.6
3	170	clear	2.9
4	173	clear	2.2
5	174	clear	2.4
6	176	clear	3.5
7	177	clear	6.4
8	181	clear	0.7
9	184	Invasion into peritoneum	3.9
10	188	clear	1.5
11	198	clear	4.9
12	201	clear	4.7
13	201	Invasion into peritoneum and into pelvic muscle	6.9
14	210	Vas deferens/ peritoneum invasion	3.5
15	211	Lymph node metastasis	2.6
16	226	clear	3.9
17	229	clear	5.3
18	230	Invasion into pelvic muscle	2.9
19	231	Invasion into pelvic muscle	7
20	237	Invasion into pelvic muscle	4.8
21	237	clear	2.2
22	241	clear	4.9
23	243	Invasion into bladder muscle	2.9
24	246	Invasion into bladder muscle	1.8
25	247	clear	5.1
26	248	Invasion into peritoneum	3
27	252	clear	2.4
28	266	Lymph node metastasis+invasion	4.9
29	275	Lymph node metastasis+invasion	6.5

Supplementary Table 1. List of age, tumour weight and metastatic status of mice used
