# Supplementary material for: Plexin-B1 Mutation Drives Metastasis in Prostate Cancer Mouse Models
Source: Cancer Res Commun. 2023 Mar 16;3(3):444–58. doi: 10.1158/2767-9764.CRC-22-0480 (PMC10019359; doi:10.1158/2767-9764.CRC-22-0480)
Supplement: Table ST2 — List of antibodies used [file crc-22-0480-s13.docx]

| **Antibody** | Source | Supplier | IHC | WB |
| --- | --- | --- | --- | --- |
| Anti-PlexinB1 | rabbit | Santa Cruz Biotechnology, Heidelberg, Germany, sc-25642 | 1:2000 | 1:500 |
| Anti-pan-cytokeratin (AE1/AE3) | mouse monoclonal | Santa Cruz Biotechnology sc-81714 | 1:250 | N/A |
| Anti-androgen receptor (N-20) | rabbit | Santa Cruz Biotechnology, sc-816 | 1:200 | N/A |
| Anti-PTEN | rabbit | Cell Signalling Technology, Leiden, The Netherlands, 9559 | 1:200 | N/A |
| Anti-phospho-MLC2 Ser19 | rabbit | Cell Signalling Technology, 3671 | 1:100 | N/A |
| Anti-β-actin | mouse monoclonal | Sigma-Aldrich, Poole, Dorset, UK | N/A | 1:12000 |
| Anti-Ki-67 | mouse monoclonal | Cell Signalling Technology, 9449 | 1:400 | N/A |

**Supplementary Table 2. List of antibodies used, their suppliers and dilution used for immunohistochemistry (IHC) and western blotting (WB).**
